# Assessing model mismatch and model selection in a Bayesian uncertainty quantification analysis of a fluid-dynamics model of pulmonary blood circulation

**DOI:** 10.1098/rsif.2020.0886

**Published:** 2020-12-23

**Authors:** L. Mihaela Paun, Mitchel J. Colebank, Mette S. Olufsen, Nicholas A. Hill, Dirk Husmeier

**Affiliations:** 1 School of Mathematics and Statistics, University of Glasgow, Glasgow, G12 8QQ, UK; 2 Department of Mathematics, North Carolina State University, Raleigh, NC 27695, USA

**Keywords:** uncertainty quantification, model mismatch, model selection, MCMC, Gaussian processes, pulmonary circulation

## Abstract

This study uses Bayesian inference to quantify the uncertainty of model parameters and haemodynamic predictions in a one-dimensional pulmonary circulation model based on an integration of mouse haemodynamic and micro-computed tomography imaging data. We emphasize an often neglected, though important source of uncertainty: in the mathematical model form due to the discrepancy between the model and the reality, and in the measurements due to the wrong noise model (jointly called ‘model mismatch’). We demonstrate that minimizing the mean squared error between the measured and the predicted data (the conventional method) in the presence of model mismatch leads to biased and overly confident parameter estimates and haemodynamic predictions. We show that our proposed method allowing for model mismatch, which we represent with Gaussian processes, corrects the bias. Additionally, we compare a linear and a nonlinear wall model, as well as models with different vessel stiffness relations. We use formal model selection analysis based on the Watanabe Akaike information criterion to select the model that best predicts the pulmonary haemodynamics. Results show that the nonlinear pressure–area relationship with stiffness dependent on the unstressed radius predicts best the data measured in a control mouse.

## 1. Introduction

Computational haemodynamics models are emerging as powerful tools for analysing cardiovascular disease progression and the effects of treatments [1] by providing essential haemodynamic metrics which could not be obtained from *in vivo* experiments [2]. The ultimate goal is achieving personalized medicine, to allow patient-specific care and treatment. Before using the models for decision-making in the clinic, they must be calibrated and fitted to data, and their credibility rigorously tested by modelling all sources of uncertainty using statistical analysis.

The current study assesses the health of the pulmonary system by integrating imaging data (obtained with micro-computed tomography (CT)), blood pressure data (measured invasively via catheterization) and blood flow data (measured with ultrasound), using a one-dimensional (1D) fluid-dynamics model combined with statistical inference. Predictions of blood pressure, blood flow and vessel area are computed in an arterial network model constructed from micro-CT images from a control mouse, and the pressure predictions are compared to dynamic data in the main pulmonary artery (MPA).

We highlight the importance of determining the uncertainty when calibrating the model to the data. Our analysis includes the uncertainty in the model parameters (which are naturally variable), in the model form/structure (the discrepancy between the model and the reality), in the measurements (the noise model), and in the simulator output (e.g. the errors from numerically integrating the model equations).

Several previous studies [3–7] have developed 1D fluid-dynamics models predicting pulmonary blood flow and pressure. However, only a few [3,4] have aimed at devising subject-specific predictions by estimating model parameters. These studies minimize the least-squares error between the model output and the measurements. While these investigations provided valuable insight into the physiology, they ignored an essential source of uncertainty resulting from the inadequacy of the model form. Our work shows that the consequence of ignoring model discrepancy is biased haemodynamic predictions and parameters, and thus inability of reliably using these models in the clinic.

### 1.1. Model parameters and types

Similar to previous studies [3,4], the 1D models analysed here have two types of parameters: specifying the vessel network (radius, length and the connectivity of arteries) and the haemodynamics (pressure and flow). The current study focuses on inferring and analysing parameters intrinsic to the haemodynamic model, including the vessel stiffness and parameters specifying the micro-circulation (boundary conditions via three-element Windkessel models attached at the terminal vessels), in a fixed network. We examine several alternative models to select the model that best predicts the data, which is performed using statistical model selection criteria based on the Watanabe Akaike information criterion (WAIC) [8]. We compare two constitutive equations: a linear and a nonlinear wall model relating vessel pressure and area. We investigate if the vessel stiffness is (a) constant over the entire network (as suggested in [9]), (b) increases with decreasing radius (as suggested in [10]), or (c) should be estimated for each vessel separately. In addition, we estimate micro-circulation parameters by introducing global scaling factors for the boundary condition parameters at the terminal arteries [4,11].

### 1.2. Bayesian inference

Due to the limited data and the model complexity, these parameters may be highly correlated or unidentifiable, thus it may be unfeasible to estimate all parameters uniquely. To address these issues, we use a Bayesian approach, with the aim to obtain the posterior distribution of the parameters [11,12]. This is analytically intractable, so we use Markov chain Monte Carlo (MCMC) to sample parameters approximately from the posterior distribution (with an asymptotic convergence guarantee). The posterior parameter samples are then used to estimate the uncertainty of model predictions throughout the pulmonary arterial network.

### 1.3. Model mismatch

Several recent studies have incorporated Bayesian parameter estimation [3,4,13–15] and uncertainty quantification (UQ) [16,17], however, most of these studies ignore the model mismatch. Our current study assumes that the model mismatch stems from two sources: (1) inadequate mathematical model (i.e. model discrepancy, since the mathematical model is not a perfect representation of the real system) [18] and (2) incorrect noise model (i.e. erroneously assuming independence when the errors are, in fact, correlated). A few studies have discussed the importance of allowing for model discrepancy. In an electrophysiology model, Lei *et al.* [19] incorporate the model discrepancy using GPs and autoregressive–moving-average (ARMA) models; the authors show using synthetic studies that ignoring the model form uncertainty leads to biased predictions and uncertainty underestimation. Additionally, Whittaker *et al.* [20] and Mirams *et al*. [21] discuss model discrepancy in a review of cardiac model calibration. Furthermore, a few studies have investigated the impact of making the wrong assumption for the measurement errors. For example, Konukoglu *et al.* [22] included an inhomogeneous variance, informed by the authors’ experience with the data, in an electrophysiology model, finding that the noise model greatly influences the inference results. Despite these findings, most cardiovascular modelling studies do not account for model mismatch [23,24].

To investigate the importance of accounting for model mismatch, we employ a Bayesian approach quantifying the uncertainty in the mathematical and the noise model form based on data. The model mismatch is explicitly modelled using Gaussian processes (GPs) [25] following an approach by Kennedy *et al.* [18]. Our Bayesian inference framework uses MCMC to jointly sample the mathematical model parameters and the model mismatch (error model) parameters from their posterior distribution. Thus, uncertainties associated with parameters, model form and measurements are all accounted for in our analysis [26].

We use physiological and synthetic pressure data to examine the consequence of inferring parameters when suspected model mismatch is unaccounted for. Results show that ignoring model mismatch biases parameter estimates and underestimates uncertainty in parameter and output space, whereas our proposed method corrects this bias. In addition, we carry out a synthetic study displaying the effect of using data from multiple vessels on the parameter inference and UQ.

Finally, we perform model selection based on WAIC to discriminate between the two constitutive models, with a number of parameter constraints related to the vessel stiffness.

## 2. Data

### 2.1. Physiological data

**Figure 1.**
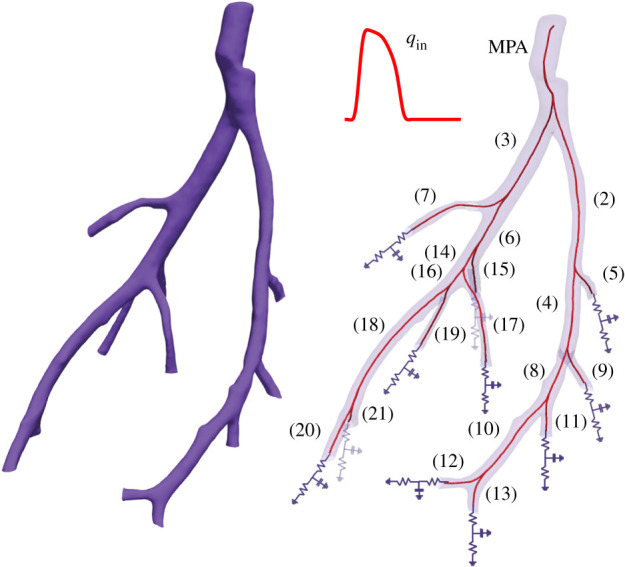
3D smoothed segmented network from a micro-CT image of a healthy mouse lung (left) and the directional graph of the same network with vessel numbers attached (right). At the network inlet, we specified a flow waveform taken from measurements (see §2.1 for a description of the experimental data), and at the outlet of each terminal vessel, we attached a three-element Windkessel model with two resistors and a capacitor.

This study compares model predictions to measured MPA blood pressure data from a control mouse lung (figure 1). The experimental protocols used to extract the haemodynamic and image data are summarized in our recent study [4], and a more detailed experimental protocol is found in [27,28]. We provide a brief overview of the data used in this study.

A 3D segmentation of the vessel geometry is obtained from micro-CT images of excised mice lungs as described in detail by Vanderpool *et al.* [28]. The image data are segmented, and using the Vascular Modeling ToolKit (VMTK,^1^ [29]) and custom algorithms discussed in [3] we obtain a 1D directional graph of the large vessels, perfusing each of the lung lobes. In this study, we assume that all vessels are straight, i.e. that they do not taper along their length. While many studies [10] examining flow in the systemic arteries account for vessel taper, the vessel radii in the large pulmonary arteries of mice show negligible inter-vessel tapering likely because the pulmonary tree is formed by rapidly branching vessels.

Dynamic pressure and flow waves were measured in the MPA. Pressure was measured using a 1.0-F pressure-tip catheter (Millar Instruments, Houston, TX) and recorded on a haemodynamic workstation (Cardiovascular Engineering, Norwood, MA) at 5 kHz. MPA flow velocity was simultaneously measured during catheterization on the same workstation via ultrasound (Visualsonics, Toronto, Ontario, CA) at a rate of 30 MHz [27]. The haemodynamic data analysed in this study include wave forms averaged over 20 cardiac cycles, using available ECG as a fiducial point.

### 2.2. Synthetic data

We also use synthetic data obtained from a forward simulation of the mathematical model. We generated synthetic, error-free data in all 21 vessels using the linear wall model and a radius-dependent exponential stiffness with parameter values consistent with the physiological data. To these data, we added non-stationary, additive Gaussian correlated errors generated using the same error parameters for all the vessels, assuming that the pressure transducer produces measurement noise that is independent of the measurement location (see §5.2 for numerical details). This error correlation induces a model mismatch if the standard assumption of independent and identically distributed (iid) measurement errors is made, i.e. if the wrong noise model is used. To make the synthetic data physiologically realistic, we ensured a signal-to-noise ratio of approximately 100 (see fig. 5 in [30]) and that the pressure monotonicity constraint is satisfied, i.e. that the pressure decreases as it approaches the periphery [31]. The mathematical model provides predictions satisfying the pressure monotonicity constraint. However, when adding noise, this is no longer guaranteed to hold. Therefore, to satisfy the monotonicity condition, the noise was constrained by using a rejection mechanism, i.e. any noise instantiation leading to a constraint violation was discarded. This mimicks the experimental procedure by which data that appear physiologically unrealistic are disposed of.

## 3. Model

### 3.1. Fluid-dynamics model of the pulmonary circulation

This study uses our previously developed 1D fluid-dynamics model [4], predicting pressure, flow and cross-sectional area explicitly in the large pulmonary arteries (shown in figure 1). For each vessel, the 1D model is derived under the assumptions that blood is incompressible and that the flow is Newtonian, laminar and axisymmetric, and has no swirl. Under these assumptions, the Navier–Stokes equations describing conservation of mass and momentum are expressed by

3.1 $$\begin{array}{rl} & \;\frac{\partial A}{\partial t}+\frac{\partial q}{\partial x}=0,\\ & \;\frac{\partial q}{\partial t}+\frac{\partial }{\partial x}(\frac{q^{2}}{A})+\frac{A}{\rho }\frac{\partial \;p}{\partial x}=-\frac{2\pi \mu r}{\rho \delta }\frac{q}{A}, \end{array}\}$$

where *x* (cm) and *t* (s) denote the spatial and temporal coordinates, and $p=\tilde{\;p}/\text{conv}$ (mmHg) denotes pressure ($\tilde{p}\;(\text{g}\cdot {\mathrm{cm}}^{-1}\;{\text{s}}^{-2})$ is in cgs units and conv = 1333.22 (mmHg (g ⋅ cm^−1^ s^−2^)^−1^) is a conversion factor). *ρ* = 1.055g ml^−1^ is the blood density and *μ* = 0.049 g cm^−1^ s^−1^ is the blood viscosity, assumed constant. Vessel radius and cross-sectional area are *r*(*x*, *t*) (cm) and *A*(*x*, *t*) = *πr*(*x*, *t*)^2^ (cm^2^), respectively. The volumetric flow rate $q=A{\bar{u}}_{x}$ (ml s^−1^) is derived assuming a Stokes boundary layer velocity profile

3.2 $$u_{x}(r,x,t)=\left\{ \begin{array}{ll} {\bar{u}}_{x},\; & \;0\leq r\leq \delta ,\;\\ {\bar{u}}_{x}\frac{\left(R-r\right)}{\delta },\; & \;\delta \leq r\leq R(x,t),\; \end{array}\right.$$

where ${\bar{u}}_{x}$ is the average velocity, $\delta =\sqrt{\mu T/2\pi \rho \;}\left(\mathrm{cm}\right)$ is the boundary layer thickness and T (s) is the length of the cardiac cycle.

To close the system of equations, we add a constitutive pressure–area relation. We investigate two wall models: a linear elastic model [32]

3.3 $$p=\frac{4}{3}\chi \left(\sqrt{\frac{A}{A_{0}}}-1\right),$$

where *χ* = *Eh*/*r*_0_ is the wall stiffness, *E* g cm^−1^ s^−2^ is Young’s modulus in the circumferential direction, *h* (cm) is the wall thickness, and *r*_0_ (cm) is the unstressed vessel radius ($A_{0}=\pi r_{0}^{2}$); and an empirical nonlinear wall model [4] given by

3.4 $$p=\chi \tan\left[\frac{\pi }{\gamma }\left(\frac{A}{A_{0}}-1\right)\right],$$

where *γ* > 0 (dimensionless) is a scaling parameter specifying the maximal lumen area *A*_∞_ for *p* → ∞, and *χ* > 0 (g cm^−1^ s^−2^) is a material parameter (defined similarly to *Eh*/*r*_0_) characterizing the half-max compliance pressure.

To investigate the claim of medial thickening and stiffening as arteries get smaller [33], we express the dependence of *χ* on *r*_0_ via the equation

3.5 $$\chi (r_{0},f_{1},f_{2},f_{3})=f_{1}\exp(f_{2}r_{0})+f_{3},$$

*f*_1_ (g cm^−1^ s^−2^), *f*_2_ (cm^−1^) and *f*_3_ (g cm^−1^ s^−2^) are constant parameters [10].

At the inlet to the network (shown in figure 1), we specify the flow (taken from measurements). Similar to previous studies [3,5,10], we assume flow conservation and pressure continuity

3.6 $$\begin{array}{rl} & \;p_{p}(L,t)=p_{d_{1}}(0,t)=p_{d_{2}}(0,t)\\ \text{and}\;\; & \;q_{p}(L,t)=\sum_{i=1}^{2}q_{d_{i}}(0,t), \end{array}\}$$

where *p* denotes the parent vessel, *d*_1_ and *d*_2_ are the daughter vessels, and *L* (cm) is the vessel length.

The micro-circulation is represented by a three-element Windkessel model (an RCR circuit) relating pressure and flow as

3.7 $$\begin{array}{rl} & \;\frac{\text{d}p(L,t)}{\text{d}t}-R_{1}\frac{\text{d}q(L,t)}{\text{d}t}=\\ & \;q(L,t)\left(\frac{R_{1}+R_{2}}{R_{2}C}\right)-\frac{\;p(L,t)}{R_{2}C}, \end{array}\}$$

where *R*_1_, *R*_2_ (g ml^−1^ cm^−1^ s^−1^) are resistances, and $C\;({\mathrm{ml}}^{-1}\;{\mathrm{cm}}^{-1}\;s^{-2}\;g^{-1}$) is the capacitance.

### 3.2. Model parameters

The haemodynamic model has three types of parameters specifying blood characteristics, vessel tissue properties and micro-vasculature dynamics. Blood viscosity and density values are taken from the literature [34] and assumed constant. The stiffness in large vessels can be measured *ex vivo* via stress–strain testing. Finally, micro-vasculature parameters are prescribed in each terminal vessel’s boundary condition.

In this study, we infer both wall model and boundary condition parameters. The linear wall model (3.3) has three parameters *f*_1_, *f*_2_, *f*_3_ characterizing the large vessel stiffness, while the nonlinear wall model (3.4) has four parameters *γ*, *f*_1_, *f*_2_, *f*_3_.

Three boundary condition parameters (*R*_1_, *R*_2_, *C*) are specified for each terminal vessel. The network in figure 1 has 11 terminal vessels giving a total of 33 parameters. Since we only have data in the MPA, these parameters are not practically identifiable. To reduce parameter dimensionality, we introduce factors (*ψ*_1_, *ψ*_2_, *c*) [4] scaling the nominal (initial) Windkessel parameters by

3.8 $$R_{1}^{j}=\psi_{1}R_{01}^{j},\;R_{2}^{j}=\psi_{2}R_{02}^{j}\;\text{and}\;C^{j}=cC_{0}^{j},$$

where $R_{01}^{j},R_{02}^{j},C_{0}^{j}$ are the nominal values for the *j*^th^ terminal vessel, computed using the junction conditions and Poiseuille’s flow, as described in detail in our previous studies [3,4]. $R_{1}^{j},R_{2}^{j},C^{j}$ are the adjusted (estimated) values for the *j*^th^ terminal vessel. *ψ*_1_, *ψ*_2_, *c* are the scaling factors, common to all terminal vessels. The scaling factors are estimated from the available data.

Forward simulations were run for both the linear and nonlinear model to ensure physiologically plausible parameter bounds. Stiffness bounds ensure pressure sensitivity to changes in *χ*, while scaling factor bounds were constructed to ensure physiological pressures (12 ≤ max (*p*) ≤ 35 mmHg). These parameters are constrained in a univariate sense, but the parameters’ behaviour in the joint space is unknown prior to carrying out the statistical analysis. Table 2 shows the univariate parameter ranges.

### 3.3. Overview of models: physiological hypotheses and model mismatch scenarios

Table 1 outlines the models considered in our work, which help explore several physiological hypotheses and model mismatch scenarios.

**Table 1.** Models investigated: two constitutive models relating pressure–area (linear (equation (3.3)) and nonlinear (equation (3.4))) with several stiffness relations: constant (*f*_1_ in equation (3.5) is 0 and all vessels share one common *f*_3_ value), vessel-specific (*f*_1_ in equation (3.5) is 0 and every vessel has its own *f*_3_ value), or radius-dependent (expressed via equation (3.5)); and model and measurement error assumptions via including or ignoring model mismatch, described in detail in §4.2. For the nonlinear wall model, we do not consider the no model mismatch scenario based on conclusions drawn from the linear model, clearly supporting modelling the model mismatch. In addition, the vessel-specific stiffness scenario is not pursued due to the interaction between the parameters *χ* and *γ* in equation (3.4), requiring vessel-specific (*χ*, *γ*). This would lead to a very large number of parameters being estimated, requiring extremely high computational efforts (simulations would most likely take months to complete).

vessel wall model	vessel stiffness type	allow for model mismatch	model abbreviation
linear	constant	no	A
	constant	yes	B/C
	radius-dependent	no	D
	radius-dependent	yes	E
	vessel-specific	no	F
	vessel-specific	yes	G
nonlinear	constant	yes	H
	radius-dependent	yes	I

By analysing these models, for the physiological data described in §2.1, we test

— if the vessel wall model is:
  (i) linear (equation (3.3)), and
  (ii) nonlinear (equation (3.4))

— if the vessel stiffness:
  (i) is constant, thus shared between the vessels (*f*_1_ in equation (3.5) is 0 and all vessels share one common *f*_3_ value),
  (ii) is vessel-specific, thus independent of the vessel (*f*_1_ in equation (3.5) is 0 and every vessel has its own *f*_3_ value), and
  (iii) is radius-dependent (expressed via equation (3.5))

— if the model mismatch, described in detail in §4.2, should be accounted for (no/yes).

## 4. Statistical methods

### 4.1. Data likelihood

We assume normally distributed errors (an assumption which we have checked by comparing the distribution of the residuals to a normal distribution, see electronic supplementary material, S4), and explore both iid errors and correlated errors. Under these assumptions, we express the likelihood function as:

— Iid errors: $y(t)∼MVN(m(\theta ,t),\sigma^{2}I)$ (multivariate normal distribution), i.e.
  4.1 $$p(y|\theta ,\sigma^{2})={\left(\frac{1}{\sqrt{2\pi \sigma^{2}}}\right)}^{n}\exp\left(-\frac{\sum_{i=1}^{n}{\left(y(t_{i})-m(\theta ,t_{i})\right)}^{2}}{2\sigma^{2}}\right),$$
  where
  4.2 $$\sum_{i=1}^{n}{\left(y(t_{i})-m(\theta ,t_{i})\right)}^{2}={\left(y(t)-m(\theta ,t)\right)}^{T}(y(t)-m(\theta ,t))$$
  is the Euclidean distance, and **y**(**t**) = (*y*(*t*_1_), …*y*(*t*_*n*_)) is the *n*-vector of temporal measurements, m(θ,t) is the *n*-vector of temporal pressure predictions from the mathematical model evaluated with parameters ***θ***, and *σ*^2^ is the measurement noise variance.
— Correlated errors: y(t)∼MVN(m(θ,t),C), i.e.
  4.3 $$\begin{array}{rl} & \;p\left(y|\theta ,C\right)=\det{\left(2\pi C\right)}^{-\frac{1}{2}}\\ & \;\exp\left(-\frac{1}{2}{\left(y(t)-m(\theta ,t)\right)}^{T}C^{-1}(y(t)-m(\theta ,t))\right), \end{array}$$
  where
  4.4 $${\left(y(t)-m(\theta ,t)\right)}^{T}C^{-1}(y(t)-m(\theta ,t))$$
  is the Mahalanobis distance, and **C** is the covariance matrix of the errors.

### 4.2. Model mismatch

The model mismatch function can be visualized by plotting the residuals, i.e. the difference between the partial differential equation (PDE) predictions and the measurements in time, see figure 8 (bottom right corner). A clear pattern is observed, indicating a correlation between the residuals. Such a plot should be used to decide on the appropriate error assumption.

When the model mismatch is neglected, the statistical model equation is equivalent to equation (4.1) and can be expressed as

4.5 $$y(t)=m(\theta ,t)+u(t)\;\text{and}\;u(t)∼MVN(0,\sigma^{2}I),$$

where *σ*^2^ is the error parameter (known as measurement noise variance).

When incorporating the model mismatch with GPs [18] (details in electronic supplementary material, S2), the statistical model equation is equivalent to equation (4.3) and is given by,

4.6 $$\begin{array}{rl} & y(t)=m(\theta ,t)+\Gamma (t)=m(\theta ,t)+f(t)+u(t)\\ \text{and}\; & f(t)∼GP(0,K|\eta ),\;u(t)∼MVN(0,\sigma_{n}^{2}I), \end{array}\}$$

where Γ(t) is the model mismatch function, **f(t)** is a latent function, GP(0,K|η) is a GP with zero mean and covariance matrix **K**, which is a function of the error parameters ***η*** (known as covariance function hyperparameters), and $\sigma_{n}^{2}$ is the residual noise variance. In equation (4.3), $C=K+\sigma_{n}^{2}I$.

A neural network covariance function with hyperparameters *w* and *b* [25] is used to fit the GP to the residuals exhibiting non-stationarity (figure 8). Equation (4.5) is a limiting case of equation (4.6) by removing the GP contribution term, **f(t)**, and setting $\sigma_{n}^{2}=\sigma^{2}$ (details in electronic supplementary material, S3).

The model mismatch Γ(t) stems from two sources: (i) the model discrepancy between the real system and the mathematical model and (ii) the incorrect noise model (i.e. making the iid assumption for correlated measurement errors), thus

4.7 Γ(t)=ζ(t)+ϵ(t),

where ζ(t) is the model discrepancy function, and ϵ(t) is the noise model function. Note that in the present article, we distinguish between *model discrepancy* and *model mismatch* in the way described above, so these words are not used synonymously.

Since this is a retrospective data analysis study, it was intrinsically impossible to separate the contributions from the measurement error (noise model) and model error (model discrepancy), which are therefore modelled with one single GP, see §8 for a more thorough discussion on this.

Possible causes for the measurement error correlation are: the temporal nature of the data (measurements at the current time point depend on measurements at previous time points), and smoothing and averaging of the data. Possible causes for the model discrepancy are: numerical errors (e.g. numerical integration of the PDEs), model assumptions (e.g. purely elastic vessel walls, or the 1D model simplification), uncertainty of the network geometry (kept fixed), and inconsistency between network geometry and haemodynamic data, which come from different mice.

### 4.3. Prior distributions

#### 4.3.1. Biophysical parameters

*Constant or radius-dependent stiffness models.* For all models with constant or radius-dependent stiffness (table 1), we used a rescaled beta distribution for the biophysical parameters to ensure positive support within physiologically realistic ranges ([*l*_*i*_, *u*_*i*_]) [4,9], *θ*_*i*_ ∼ rescaled beta(1, 1), *l*_*i*_ ≤ *θ*_*i*_ ≤ *u*_*i*_, where i=1,…k, with *k* being the parameter dimensionality.

**Figure 2.**
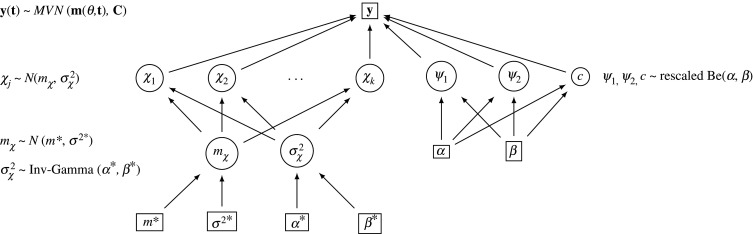
Bayesian hierarchical model used for the vessel-specific stiffness analysis. The data, denoted by **y**(**t**), are assumed to follow a multivariate normal distribution with mean **m**(***θ***, **t**) and covariance matrix **C**. If iid errors are assumed (i.e. the model mismatch is ignored), **C** is a diagonal matrix, **C** = *σ*^2^**I** (where *σ*^2^: noise variance and **I**: identity matrix), and if correlated errors are assumed (i.e. the model mismatch is incorporated), **C** is a full matrix. The biophysical parameters, ***θ*** = (*χ*_1_, …*χ*_*k*_, *ψ*_1_, *ψ*_2_, *c*) (described in §3.2), and the hyperparameters, $m_{\chi },\sigma_{\chi }^{2}$, are *a priori* drawn from the distributions indicated in the graphical model. The circle represents variable quantities, which are inferred using MCMC, and the rectangle stands for fixed quantities. Inference in this model is analytically intractable, and we resort to a Gibbs sampling scheme, discussed in electronic supplementary material, equations (24)–(26). Note that a modification of this model, where an additional edge is introduced from $\sigma_{\chi }^{2}$ to $m_{\chi }$ allows these two parameters to be integrated out in closed form, potentially leading to a more efficient sampling scheme; however, these equations are less intuitive, so the details have been relegated to electronic supplementary material, S5.

*Vessel-specific stiffness in a Bayesian hierarchical model.* Different pulmonary arteries may have different vessel wall stiffness values, but since all vessels have similar tissue composition, the parameters are related. A Bayesian hierarchical model [35] is needed to incorporate our prior notion that the vessel stiffnesses are similar, and this model provides a mechanism of information sharing among the vessel stiffness parameters. The dependence of the stiffness parameters can be captured by using a common ‘population’ prior distribution, from which vessel-specific stiffness parameters are sampled. Next, to allow the stiffness parameters to influence each other, we introduce a layer of priors for the hyperparameters of the population distribution. This construct enables the hyperparameters to be variable, ensuring a dependency between the stiffness parameters, and the hyperparameters’ uncertainty is naturally modelled. The result is a Bayesian hierarchical model (shown in figure 2), which tends to avoid overfitting the existing data by allowing information sharing between the stiffness parameters. This model subsumes two simpler models as limiting cases: the model where all vessels have the same stiffness (when the prior distribution of the vessel-specific stiffness parameter collapses to a delta spike), and the model of independent vessel-specific stiffness parameters without information sharing (when the prior distribution of the stiffness parameters is the uniform distribution). Electronic supplementary material, S5 offers more details of the Bayesian hierarchical model.

Under this model (figure 2), the three Windkessel parameters *ψ*_1_, *ψ*_2_, *c* are assumed common to all the vessels, and to ensure positive support (within physiological ranges) for them, we used a rescaled beta distribution.

#### 4.3.2. Error parameters

For the analysis neglecting the model mismatch (i.e. assuming iid errors, see equations (4.1) and (4.5)), we place a conjugate weakly informative inverse-gamma prior on the error parameter, *η* = {*σ*^2^}: $\sigma^{2}∼IG(a,b)$, with *a* = 0.001 and *b* = 0.001, leading to an IG posterior distribution.

For the model mismatch analysis (i.e. assuming correlated errors, see equations (4.3) and (4.6)), the hyperparameters of the GP neural network covariance function ***η*** = {*w*, *b*}, are given a log uniform distribution with the range chosen based on maximizing the profile log likelihood (see electronic supplementary material, S8.4 for details).

### 4.4. Posterior inference with Bayesian methods

The posterior distribution is computed as

4.8 p(θ,η|y)∝p(y|θ,η)p(θ,η),

where ***θ*** are the biophysical parameters and ***η*** are the error parameters. In this study, we pursue Bayesian inference based on sampling the biophysical and error parameters from their posterior distribution (equation (4.8)), see electronic supplementary material, S6 for details.

### 4.5. Bayesian model selection: WAIC

WAIC [8] is used for model selection as its computation is straightforward from the MCMC posterior samples (see electronic supplementary material, S1.1 for the mathematical details). Out of a number of candidate models, the model which registers the lowest WAIC score is best supported by the data.

## 5. Simulations

### 5.1. Code

Our statistical methods were implemented in Matlab (Mathworks, Natick, MA) and simulations were run on a RedHat Enterprise Linux 6 machine with Intel(R) Xeon(R) CPU E5-2680 v. 2 2.80GHz and 32GB RAM. The simulated pressure waveforms were obtained by numerically solving the PDEs in equations (3.1)–(3.7) using a two-step Lax–Wendroff scheme [36] implemented in $C^{++}$ by Olufsen *et al.* [4,10], which is second-order accurate in space and time. A spatial and temporal discretization of Δ*x* = 0.025 (mm) and Δ*t* = 1.34 × 10^−5^ (s) were used after testing different discretizations. This satisfied the Courant–Fredrich–Levy (CFL) condition and ensured minimal numerical error. We implemented the GP models using the GPstuff toolbox [37] and the MCMC convergence dignostics using the MCMC toolbox [38].

### 5.2. Set-up

We split the simulations into two categories. The simulations in the first category use the measured MPA pressure data for all the models summarized in §3.3 and tables 1 and 2. Simulations in the second category use synthetic data (20 data instantiations) generated in 1, 3 or 21 vessels from the linear wall model with radius-dependent stiffness and correlated errors (model E in table 2) created using a GP with a neural network kernel.

**Table 2.** Models analysed for the measured data: the constitutive models (linear and nonlinear) with model parameters (*f*_1_, *f*_2_, *f*_3_, *γ*, *ψ*_1_, *ψ*_2_, *c*) prior ranges. We indicate whether the model mismatch is incorporated (by yes or no), and if it is, the hyperparameters *w* and *b* for the GP model mismatch are given. In the right-most column, the symbol ‘+’ indicates that the emulation approach was used to accelerate the MCMC simulations, while ‘−’ indicates that the standard MCMC was used. The stiffness relation used is given in equation (3.5).

model abbreviation	model	no.parameters	*f*_1_ ( ×10^3^)	*f*_2_	*f*_3_ ( ×10^4^)	*γ*	*ψ*_1_	*ψ*_2_	*c*	model mismatch	*w* ( ×10^4^)	*b*	emulator
A	linear	4	0	not used	(2, 10)	−	(0.05, 2.50)	(0.05, 2.50)	(0.05, 2.50)	no	−	−	+
B	linear	6	0	not used	(2, 10)	−	(0.05, 2.50)	(0.05, 2.50)	(0.05, 2.50)	yes	(1, 9)	(1, 500)	+
C	linear	6	0	not used	(2, 10)	−	(0.05, 2.50)	(0.05, 2.50)	(0.05, 2.50)	yes	(1, 9)	(1, 500)	−
D	linear	6	(1, 10^4^)	(−300, −50)	(3, 6)	−	(0.05, 2.50)	(0.05, 2.50)	(0.05, 2.50)	no	−	−	−
E	linear	8	(1, 10^4^)	(−300, −50)	(3, 6)	−	(0.05, 2.50)	(0.05, 2.50)	(0.05, 2.50)	yes	(1, 9)	(1, 500)	−
F	linear	24	0	not used	(2, 10)*	−	(0.05, 2.50)	(0.05, 2.50)	(0.05, 2.50)	no	−	−	−
G	linear	26	0	not used	(2, 10)*	−	(0.05, 2.50)	(0.05, 2.50)	(0.05, 2.50)	yes	−	−	−
H	nonlinear	7	0	not used	(3, 50)	(1,2*π*)	(0.05, 2.50)	(0.05, 2.50)	(0.05, 2.50)	yes	(1, 9)	(1, 500)	+
I	nonlinear	9	(5, 10^2^)	(-200, 0)	(1, 50)	(1,2*π*)	(0.05, 2.50)	(0.05, 2.50)	(0.05, 2.50)	yes	(1, 9)	(1, 500)	−

Legend: * in column *f*_3_ indicates that 90% prior probability has been placed on these bounds as part of a Bayesian hierarchical scheme (figure 2) to infer 21 individual vessel stiffness parameters.

### 5.3. Computational efficiency

For some of the simulations in the first category, models A, B and H, summarized in tables 1 and 2, we focus on computational efficiency, thus we implement MCMC with a GP surrogate (emulator) for the posterior distribution [12]. This approach is motivated by the high computational complexity of repeated numerical integrations of the PDEs in the Bayesian analysis. The method, described in detail in electronic supplementary material, S7, significantly speeds up computationally expensive simulations, which is essential if the analysis performed here is to be translated to human medicine and practical clinical decision support. Constructing emulators for models with large numbers of parameters poses computational challenges due to the large dimension of the input space that has to be covered, which is beyond the remit of the present paper.

## 6. Results

### 6.1. Importance of correcting for model mismatch

We compare inference results based on MCMC between the conventional method ignoring model mismatch and our proposed approach, which explicitly incorporates the model mismatch, defined in equation (4.7), with GPs. The results are shown for synthetic and physiological data. Convergence of MCMC methods was tested using the Geweke test [39] (the *p*-values from the *Z* test were greater than 0.05) and the Brooks multivariate potential scale reduction factor (MPSRF) [40] (ensuring that MPSRF ≤ 1.1).

#### 6.1.1. Synthetic data

**Figure 3.**
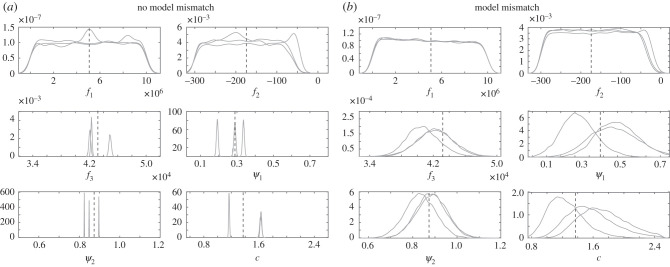
Inference results for three synthetic datasets generated from model E in table 2 with correlated errors using (*a*) the standard method, which ignores model mismatch, defined in equation (4.7), and (*b*) a GP introduced to allow for model mismatch. We show marginal posterior densities for the parameters of the exponential radius-dependent stiffness linear model (*f*_1_, *f*_2_, *f*_3_, *ψ*_1_, *ψ*_2_, *c*), where the stiffness is given by equation (3.5). The different densities per parameter correspond to three random datasets out of 20 (for complete results, see table 3). The black dashed vertical line marks the ground truth parameter values which generated these data.

We generated synthetic data using model E (table 2) with additive, correlated Gaussian errors in the MPA (20 different data instantiations), which mimics the physiological data (as described in §2.2). We then ran two MCMC simulations: one which incorporates the model mismatch, and another simulation which does not. Parameter estimates obtained from these two simulations are compared to the ground truth parameter values in table 3 using the relative sum of squared errors (SSE),

6.1 $$\sum_{i=1}^{k}{\left(\frac{\theta_{i}-{\hat{\theta }}_{i}}{\theta_{i}}\right)}^{2},$$

which is the relative deviation in Euclidean space of the estimated values from the true parameter values. We also show the median marginal and joint posterior density value of the true parameter vector, ***θ*** = (*f*_1_, *f*_2_, *f*_3_, *ψ*_1_, *ψ*_2_, *c*), under the assumed model, as found from 20 synthetic datasets. To obtain the marginal posterior density of the true parameter vector, we used the kernel smoothing function estimate for univariate data with the optimal bandwidth for normal densities [42]. To check for consistency of the results, the joint posterior density was obtained in two ways: using the multivariate kernel density estimation with the bandwidth estimated with Silverman’s rule [43], and using Chib’s method (see §2.1 in [41]). The parameters were scaled to the same order of magnitude, as both methods were affected by having parameters with different orders of magnitude. Figure 3 displaying the marginal posterior density values of the parameters for three of the 20 datasets shows that with the standard method neglecting model mismatch, the ground truth parameter value lies in the tail of the posterior distribution for most cases investigated, and the posterior uncertainty is underestimated. By contrast, with our proposed method allowing for model mismatch, the posterior distribution contains the true parameter and the posterior uncertainty is wider. For the complete set of results, we refer to table 3, which shows that neglecting model mismatch leads to a lower (better) relative SSE for the parameter estimates. However, as seen from figure 3, a small SSE does not rule out the possibility of seriously underestimating the uncertainty. A better measure, to capture both estimation accuracy and UQ, is the marginal posterior density of the true parameters. Here, we see that the median marginal posterior density value of the true parameter with the standard method is substantially lower (worse) for the identifiable parameters *f*_3_, *ψ*_1_, *ψ*_2_ and c.

**Table 3.** Results obtained when allowing for or ignoring model mismatch, defined in equation (4.7), on synthetic data generated from model E in table 2 with correlated errors. First row: standard approach ignoring model mismatch; second row: the proposed new method, where a GP mismatch model has been introduced. The relative sum of squared errors (SSE), as well as the median posterior distribution of the true parameter vector, ***θ*** = (*f*_1_, *f*_2_, *f*_3_, *ψ*_1_, *ψ*_2_, *c*), under the assumed model are presented (median calculated from 20 datasets). Marginal and joint posteriors were obtained from the MCMC samples with kernel density estimation (first entry in last column) and Chib’s method [41] (second entry in last column). Parameters were scaled to the same order of magnitude.

allow for model mismatch	SSE	median *p*(*θ*_*i*_|**y**)	median *p*(***θ***|**y**)
no	0.02	(9.8 × 10^−08^, 0.004, 0, 0, 0, 0)	0, 0
yes	0.03	(9.7 × 10^−08^, 0.004, 0.0001, 4.36, 5.25, 1.30)	1037, 648

#### 6.1.2. Physiological data

For the physiological data, table 4 shows comparative results from the MCMC analysis for the nine models explored. We present the median posterior density value (50th posterior quantile) for each of the models’ parameters, and the associated 95% posterior credible interval obtained from the 2.5% and 97.5% quantiles of the MCMC posterior samples. WAIC scores, calculated from 1000 random MCMC samples, are compared to the Euclidean distance (equation (4.2)) obtained with the median posterior parameter value. A lower WAIC score indicates a better model.

**Table 4.** Summary of the MCMC simulation results on measured data for the constitutive models considered (linear and nonlinear) with model parameters (*f*_1_, *f*_2_, *f*_3_, *γ*, *ψ*_1_, *ψ*_2_, *c*). We indicate whether model mismatch, defined in equation (4.7), is incorporated (by yes or no), and if it is, the parameters *w* and *b* for the GP model mismatch are given. In the right-most column, the symbol ‘+’ indicates that the emulation approach was used to accelerate the MCMC simulations, while ‘−’ indicates that the standard MCMC was used. We have run 5000 MCMC iterations for the models which use the emulator (models A, B and H); 300 000 for the vessel-specific stiffness models which do not use the emulator (models F and G); and 150 000 MCMC iterations for the rest of the models which also do not use the emulator (models C, D, E, I) (the emulator approach needs much fewer iterations—see the electronic supplementary material, S7 for an explanation). We indicate the median posterior distribution value, as well as the 95% credible interval based on the posterior distribution. We display the WAIC score calculated from 1000 MCMC samples and the Euclidean distance obtained based on the posterior median parameter values. The exponential stiffness relation used is given in equation (3.5). If 21 individual stiffness parameters are inferred (figure 2), marked by * in the table, we list the stiffness values in electronic supplementary material, S9.

model abbreviation	model	*f*_1_ ( × 10^5^)	*f*_2_	*f*_3_ ( × 10^4^)	*γ*	*ψ*_1_	*ψ*_2_	*c*	model mismatch	*w* ( × 10^4^)	*b*	WAIC	Euclidean distance	emulator
A	linear	0	not used	5.17 (5.09, 5.25)	−	0.21 (0.19, 0.23)	0.88 (0.88, 0.89)	1.44 (1.39, 1.49)	no	−	−	408	66	+
B	linear	0	not used	4.31 (3.91, 4.72)	−	0.28 (0.18, 0.42)	0.87 (0.73, 1.00)	1.35 (0.98, 1.96)	yes	5.36 (4.32, 7.09)	137 (102, 197)	−4515	551	+
C	linear	0	not used	4.31 (3.91, 4.71)	−	0.29 (0.18, 0.41)	0.87 (0.73, 1.00)	1.34 (0.97, 1.94)	yes	5.41 (4.27, 7.09)	138 (101, 198)	−4515	571	−
D	linear	52.6 (2.59, 97.8)	−162 (−293, −51.1)	5.17 (5.10, 5.25)	−	0.21 (0.19, 0.23)	0.89 (0.88, 0.89)	1.45 (1.40, 1.50)	no	−	−	397	64	−
E	linear	51.1 (2.80, 97.4)	−171 (−293, −56.6)	4.32 (3.91, 4.72)	−	0.29 (0.18, 0.42)	0.87 (0.74, 1.01)	1.34 (0.96, 1.93)	yes	5.39 (4.28, 7.08)	138 (102, 200)	−4515	552	−
F	linear	0	not used	*	−	2.45 (2.33, 2.50)	0.21 (0.17, 0.26)	2.08 (0.11, 2.48)	no	−	−	−107	26	−
G	linear	0	not used	*	−	0.46 (0.19, 1.05)	0.86 (0.72, 1.10)	1.12 (0.45, 1.86)	yes	4.65 (3.72, 6.25)	161 (115, 262)	−4522	242	−
H	nonlinear	0	not used	9.17 (6.66, 12.1)	5.18 (4.38, 6.07)	0.37 (0.27, 0.47)	0.94 (0.80, 1.06)	1.60 (1.15, 2.27)	yes	4.91 (3.97, 6.44)	178 (126, 286)	−4522	384	+
I	nonlinear	0.58 (0.20, 0.97)	-6.00 (-18.0, -2.13)	2.00 (1.09, 3.40)	5.09 (4.43, 6.13)	0.34 (0.24, 0.43)	0.97 (0.82, 1.09)	1.58 (1.16, 2.27)	yes	4.45 (3.62, 5.76)	196 (130, 320)	−4530	417	−

**Figure 4.**
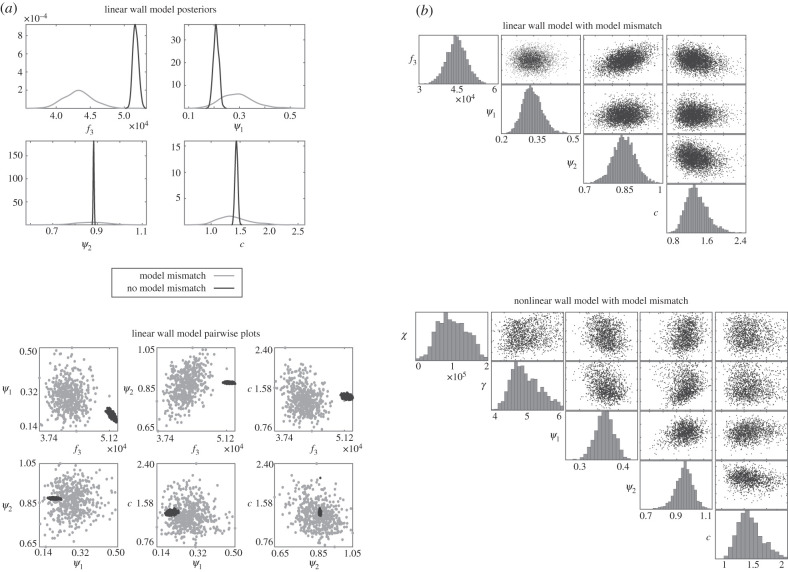
(*a*) Marginal posterior densities (top) and pairwise scatterplots (bottom) of the posterior sample (obtained with MCMC) for the constant stiffness linear wall model with the standard method ignoring the model mismatch (equation (4.7)) (black) versus our proposed GP mismatch model (grey), i.e. models A and B in table 2. (*b*) Pairwise scatterplots between the MCMC posterior parameter samples of the linear model with constant stiffness and model mismatch (top), i.e. model B in table 2, and nonlinear model with radius-dependent stiffness and model mismatch (bottom), i.e. model I in table 2. For the nonlinear model, we express *χ*(*f*_1_, *f*_2_, *f*_3_) in equation (3.5) instead of individual parameters *f*_1_, *f*_2_, *f*_3_ due to parameter identifiability issues, see §6.2 for a discussion on this. Here, we show the distribution of *χ*(*f*_1_, *f*_2_, *f*_3_) in equation (3.5) for radius *r*_0_ corresponding to vessel 1, the MPA, but the pattern of the distribution is similar for the other vessels.

**Figure 5.**
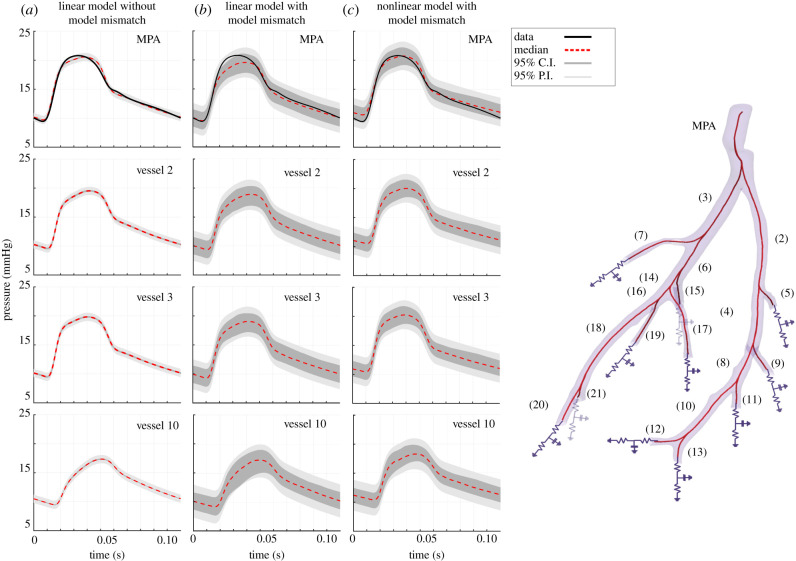
Ninety-five per cent credible intervals (C.I.) and prediction intervals (P.I.) for the pressure prediction from the linear model with one stiffness and ignoring model mismatch in equation (4.7) (left) (model A in table 2), the linear model with one stiffness and model mismatch correction (centre) (model B in table 2), and the nonlinear model with radius-dependent stiffness and model mismatch correction (right) (model I in table 2), obtained from MCMC posterior samples. We superimpose the measured pressure data in the MPA and the median prediction, and show plots in three other vessels (for all the other vessels, see electronic supplementary material, S9). This figure illustrates the predictive performance of these models (A, B, I) and helps visualize pressure profiles in parts of the vessel network for which no experimental data are available. The figure is not used to justify our model selection results, for which table 4 should be used.

Models incorporating the model mismatch, defined in equation (4.7), record a lower (better) WAIC and a higher (worse) Euclidean distance in output space compared to models which ignore it, implying that the former are better supported by the data, and that minimizing the Euclidean distance, which is equivalent to minimizing the mean squared error (MSE), is a sub-optimal inference procedure. The reason is that the MSE does not take the error correlation into account and does not penalize models for poor UQ. This is illustrated in figure 4 showing that the posterior uncertainty in parameter space is much wider when allowing for model mismatch, which aligns with findings from the synthetic study. Moreover, parameters *f*_3_ and *ψ*_1_ have different posteriors depending on whether the model mismatch is incorporated (figure 4). Additionally, figure 5 displays the posterior uncertainty in output space for pressures in several vessels using the linear model with constant stiffness ignoring or correcting for the model mismatch (models A and B in table 2). Similar to the posterior uncertainty in parameter space, the posterior uncertainty in output space is much wider when correcting for the model mismatch, which is also shown in electronic supplementary material, table S1; there we provide the time-averaged 95% explanatory and predictive credible interval width for the pressure data from every model.

### 6.2. Parameter posteriors

Figure 4 shows the posterior correlations and the marginal posterior densities for the linear models A, and B and nonlinear model I. The marginal posterior distributions have one clear mode and correlations between the parameters are negligible for the linear models. When the nonlinear model is used, we plot *χ*(*f*_1_, *f*_2_, *f*_3_) in equation (3.5) rather than the individual *f*_1_, *f*_2_, *f*_3_ parameters, since the term *f*_2_*r*_0_ is close to 0 (compare prior range for *f*_2_ in table 2 to posterior uncertainty interval in table 4), leading to unidentifiability of *f*_1_ and *f*_3_.

### 6.3. Vessel wall model

Table 4 indicates that the nonlinear wall model outperforms the linear wall model, since the former registers a lower (better) WAIC score than the latter (linear model G: −4522, nonlinear model I: −4530). More specifically, results suggest that the nonlinear model with radius-dependent stiffness (model I) is preferred, as it registers the lowest (best) WAIC score computed based on the MPA-measured pressure data.

Based on the WAIC scores in table 4, we conclude that out of all the models investigated, the best linear wall model is that with vessel-specific stiffness (model G), and the best nonlinear model is that with an exponential radius-dependent stiffness (model I), incorporating the model mismatch in both models. The nonlinear model with vessel-specific stiffness is not considered due to the interaction between the parameters *χ* and *γ* in equation (3.4), requiring vessel-specific (*χ*, *γ*). This would lead to a large number of parameters being estimated, requiring demanding computational efforts (simulations would most likely take months to complete.^2^)

### 6.4. Vessel wall stiffness

**Figure 6.**
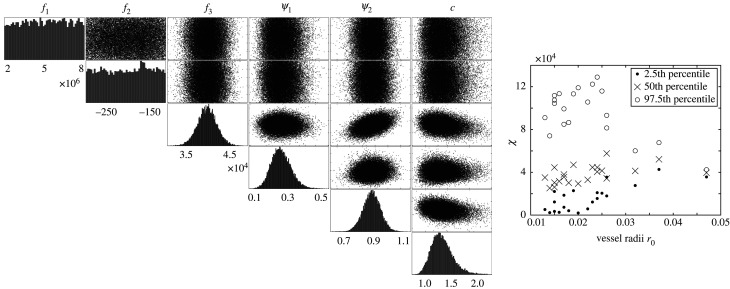
Left panel: MCMC results (marginal posterior densities and scatterplots) based on the linear wall model with exponential stiffness (equation (3.5)) and correcting for model mismatch (defined in equation (4.7)), for measured data. Right panel: MCMC results for 21 individual stiffness values corresponding to every vessel radius *r*_0_ for the linear wall model and correcting for model mismatch. Here, we show the posterior median value for each vessel stiffness parameter, and the 2.5th and 97.5th quantiles.

**Figure 7.**
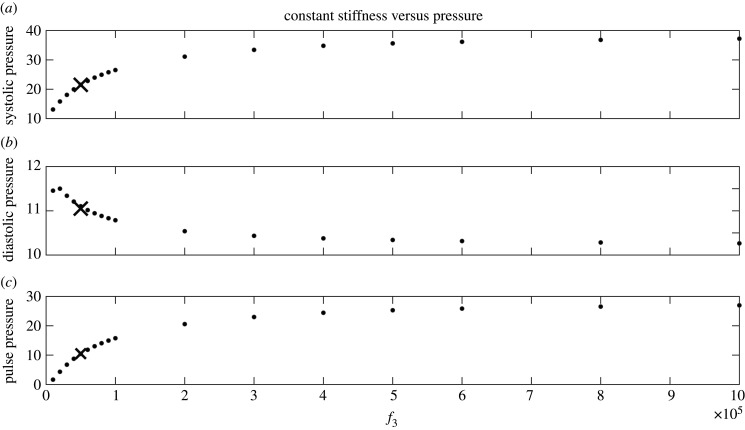
Relation between the radius-constant stiffness and the systolic, diastolic and pulse pressure for the linear wall model. Here, we vary *f*_3_ within the range [10^4^, 10^6^], *f*_1_ is set to 0, *f*_2_ can take any value in the exponential radius-dependent expression in equation (3.5), and the Windkessel parameters *ψ*_1_, *ψ*_2_, *c* are kept fixed to 0.30, 0.97, 1.23, which are plausible values for the measured data. A similar trend is observed when the Windkessel parameters are fixed to other values, or when the nonlinear wall model is used. We mark by a cross point the *f*_3_ stiffness value estimated from the measured data using the constant stiffness linear model.

Table 4 shows a lower WAIC score for the linear wall model with vessel-specific stiffness (model G) relative to the other linear models, which assume constant or radius-dependent stiffness (B, E). The exponential radius-dependent stiffness model (E) has the same WAIC score as the constant stiffness model (B), suggesting that the additional model complexity is not beneficial, as the *f*_1_ and *f*_2_ parameters are non-influential (their marginal posterior distributions are uniform on the prior range), see the left panel of figure 6. In addition, the 21 stiffness model reveals that the median posterior stiffness values are nearly all similar (right panel of figure 6). However, the stiffness becomes increasingly variable for small-radius vessels, which is also evident from the 95% credible interval width presented in electronic supplementary material, S9. This suggests that the Bayesian hierarchical model should allow for vessel-specific variance, thus the common variance $\sigma_{\chi }^{2}$ in figure 2 should be replaced by a variance-covariance matrix. This method extension will lead to a substantial increase in the computational complexity due to higher parameter space dimension, and thus, it is subject to future work. Almost all the stiffness values are of the order 10^4^, which is the regime where the MPA systolic, diastolic and pulse pressures are sensitive to changes in stiffness (figure 7). The plot is produced with a set of scaling parameters consistent with the physiological data (*ψ*_1_ = 0.30, *ψ*_2_ = 0.97, *c* = 1.23).

In addition, table 4 shows that the stiffness in the nonlinear model has only a weak dependence on the radius (model I), as expressed in equation (3.5), since the term *f*_2_*r*_0_ is close to 0 (compare prior range for *f*_2_ in table 2 to posterior uncertainty interval in table 4).

### 6.5. Model fits

**Figure 8.**
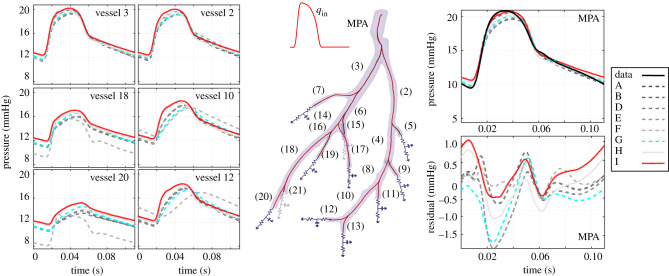
Pressure predictions obtained using the MCMC posterior samples for the parameters from all the models considered—see §3.3 and tables 2 and 4 for a summary of the models, which are denoted by A–I in the figure legend. We show the median pressure signal for seven of the 21 blood vessels in time (see electronic supplementary material, S9 for all the other vessels). We superimpose the measured pressure data in the MPA (top right). Examples of pressure residuals, that is, the difference between the predicted and measured blood pressure, are shown in the bottom right panel.

**Figure 9.**
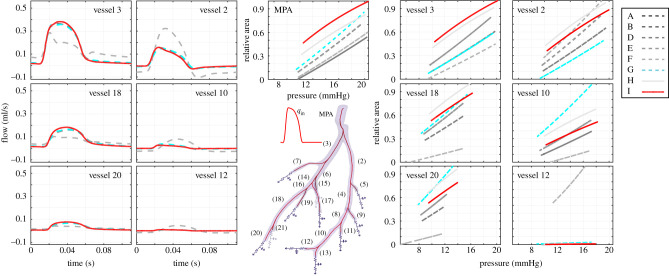
Flow (left side) and pressure–area (right side) predictions obtained using the MCMC posterior sample for the parameters from all the models considered, see §3.3 and tables 2 and 4 for a summary of the models, which are denoted by A–I in the figure legend. We show the median flow predictions and pressure versus standardized cross-sectional area predictions for seven of the 21 blood vessels (see electronic supplementary material, S9 for all the other vessels). The area, *A*_*i*_ is standardized per vessel *i* to lie between [0,1] using the expression: (*A*_*i*_ − *l*_*i*_)/(*u*_*i*_ − *l*_*i*_), where *l*_*i*_, *u*_*i*_ are the maximum and minimum area value for vessel *i*, listed in electronic supplementary material, S9.

Figures 8 and 9 show the model fits for all the models analysed. The median pressure predictions obtained using the MCMC-simulated posterior parameter values (figure 8) are qualitatively similar for all nine models investigated. Pressure predictions in the MPA are compared between all models, all producing a waveform similar to the measured data. The best linear model (model G) fits the measured data better in the diastolic phase, but gives a peak shift in the systolic phase (figure 8). On the other hand, the best nonlinear model (model I) provides a better fit in the systolic phase, but has a slight discrepancy in diastole. Generally, the pressure increases more steeply in the systolic phase for the nonlinear model compared to the linear model.

Figure 8 also shows that the pressure predicted with the nonlinear model is slightly higher than that predicted with the linear model for all 21 vessels. In addition, the predictions obtained with the linear model with 21 individual stiffnesses while ignoring model mismatch (model F) provides fits similar to the other models in the proximal arteries, but predicts downstream pressure waves with different shape and increased oscillatory behaviour. This suggests that assuming a vessel-specific stiffness and ignoring the model mismatch provides poor extrapolation performance. When analysing the median flow predictions obtained from the parameter posteriors (figure 9), we note that all are again very similar in shape, except for the outlier model F. We observe an unequal flow distribution between the right and left side of the tree for all the models except model F.

Figure 9 shows predicted pressure–area relations using the posterior median parameter values. The best nonlinear model (model I) consistently predicts larger areas than the best linear model (model G) for the proximal vessels (see predictions in MPA and vessels 2 and 3), and the opposite trend is observed for most terminal vessels (see predictions in electronic supplementary material, S9). Furthermore, we observe that the nonlinear model with constant stiffness (model H) gives systematically larger area values than the nonlinear model with radius-dependent stiffness (model I) except in vessels 1 and 3, which aligns with the former model having a smaller stiffness than the latter (table 4). The linear model with 21 individual stiffnesses without model mismatch (model F) gives drastically different results than the other models for some of the vessels (e.g. vessels 4, 8, 12 in electronic supplementary material, S9), which further indicates that this model can lead to drastic changes in downstream predictions.

### 6.6. Future experimental design

**Figure 10.**
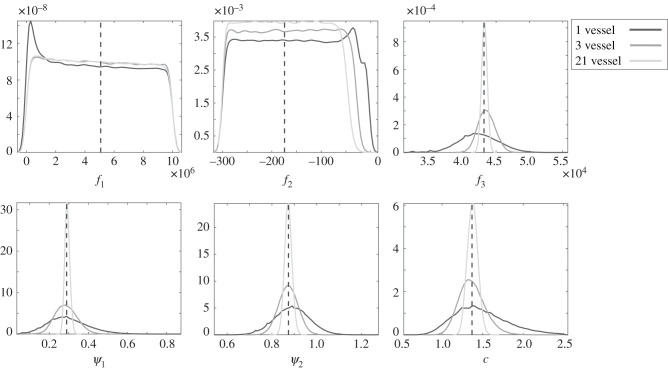
Synthetic data results obtained by agglomeration of MCMC posterior samples over 20 data instantiations. We show marginal posterior densities for the parameters *f*_1_, *f*_2_, *f*_3_, *ψ*_1_, *ψ*_2_, *c* of the linear wall model with exponential stiffness, *χ*(*f*_1_, *f*_2_, *f*_3_) given in equation (3.5). We superimpose results for three simulations: one which uses synthetic data from one vessel (MPA) for inference—dark grey line, a second one which uses data from three vessels (MPA and its two daughter vessels)—medium grey line and a third one which uses data from all 21 vessels—light grey line. The black dashed vertical line marks the ground truth parameter values which generated these data.

We test by a synthetic study if parameters *f*_1_ and *f*_2_ in model E, i.e. the linear model with exponential radius-dependent stiffness (equation (3.5)) become influential as complementary data from downstream vessels are added. We generated synthetic data from this model and, as described in §2.2, we added additive correlated Gaussian errors to them. We created 20 synthetic datasets and applied MCMC to infer the data-generating parameter values. In figure 10,we show the agglomerated MCMC posterior distributions from all 20 data instantiations, and superimpose the true parameter values (the peak of the agglomerated distributions should coincide with the true parameter values). We stress that this is purely for visualization purposes, since agglomerated results over different datasets is a non-conventional Bayesian approach. For a fully Bayesian approach, we calculate the marginal posterior distribution, as well as the joint posterior distribution of the true parameter for each of the datasets, and find the median over the datasets, shown in table 5. For the joint posterior distribution, we used the multivariate kernel density estimation. Figure 10 shows that the peak of the agglomerated distributions aligns with the ground truth parameter values for the influential parameters, validating our inference procedure. Even with data from more than one vessel (3 or 21 vessels), *f*_1_ and *f*_2_ parameters remain non-influential (close to uniform marginal posterior density). The uncertainty for all the other parameters (*f*_3_, *ψ*_1_, *ψ*_2_ and *c*) is reduced and the distributions become increasingly focused around the true parameter values with increasing complementary data. Additionally, in table 5, we quantify how the marginal and joint posterior densities values of the true parameters increase with the amount of vessel data.

**Table 5.** Inference results obtained using synthetic data, to which we added additive, correlated Gaussian error, from one vessel (MPA), three vessels (MPA and its two daughter vessels) and all 21 vessels. The model mismatch was included in the analysis, and the data were generated using the linear wall model with exponential stiffness, *χ*(*f*_1_, *f*_2_, *f*_3_), given in equation (3.5). The median marginal and joint posterior density, of the true parameter vector, ***θ*** = (*f*_1_, *f*_2_, *f*_3_, *ψ*_1_, *ψ*_2_, *c*) are presented for each of the three scenarios (median calculated from 20 datasets). Joint and marginal posteriors were computed using the MCMC samples with kernel density estimation. Parameters were scaled to the same order of magnitude.

data	median *p*(*θ*_*i*_|**y**)	median *p*(***θ***|**y**)
one vessel	(9.8 × 10^−08^, 0.004, 0.0001, 4.30, 5.22, 1.30)	2.8 × 10^+03^
three vessels	(9.9 × 10^−08^, 0.004, 0.0003, 6.78, 9.61, 2.61)	3.4 × 10^+04^
21 vessels	(9.9 × 10^−08^, 0.004, 0.001, 38.4, 27.0, 6.39)	3.9 × 10^+06^

### 6.7. Accuracy of emulator

**Figure 11.**
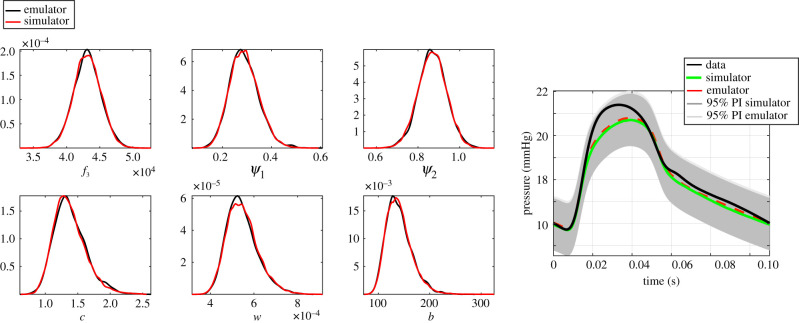
Uncertainty quantification in input (left panel) and output space (right panel) obtained with the emulation MCMC method (model B in table 2) and the standard MCMC method (model C).

Table 4 and figure 11 show that the parameter inference results obtained with the emulation approach (model B) are comparable with those obtained with the conventional method (model C), i.e. the marginal posterior densities overlap. This suggests that no bias is introduced by the emulator. This finding is further confirmed by similar results in output space between the two approaches, summarized in electronic supplementary material, table S1 and figure S11, i.e. the median pressure signals and 95% posterior predictive intervals are very similar.

## 7. Discussion

In this study, we have explored several mathematical models of the pulmonary circulation (a linear and a nonlinear wall model with different vessel wall stiffness assumptions) and two error models capturing or ignoring the model mismatch, as defined in equation (4.7). We have used Bayesian inference to find the model that can best predict the measured MPA blood pressure, while providing UQ associated with that pressure prediction. We have also tested the validity of our parameter inference procedure by a synthetic study.

### 7.1. Importance of correcting for model mismatch

Neglecting the model mismatch by obtaining point estimates based on MSE minimization biases parameter estimates and underestimates uncertainty in parameter and output space. This finding is based on synthetic data with known parameters, and tallies with results from the physiological data. The model mismatch is a consequence of wrong measurement error assumptions (i.e. iid for correlated measurement errors) and ignoring model discrepancy between the real system and the mathematical model. In this study, we proposed a method based on GPs to allow for the model mismatch, which circumvents the limitations outlined above. Figure 3 clearly illustrates that the posterior uncertainty in parameter space is under-dispersed when the model mismatch is neglected, and the true (data-generating) parameter values lie in the tail of the posterior distribution for most datasets; however, this is not the case for our proposed method of model mismatch. This is in line with results from the measured data, as evident in figure 4*a*, that also shows very narrow uncertainty bounds in parameter space and in output space (figure 5). Moreover, the model selection criteria (WAIC in table 4), clearly and consistently favours the models with model mismatch, further strengthening our statement.

We emphasize that most studies in the literature rely on minimising the Euclidean distance (i.e. MSE) in equation (4.2), which implicitly ignores the model mismatch. This approach is equivalent to maximizing the likelihood in equation (4.1) under the assumption of additive Gaussian iid errors. However, in the presence of model mismatch, the estimates that minimize the MSE are different from the estimates which maximize the likelihood in equation (4.3). Our work demonstrates that ignoring model mismatch leads to biased point estimates, and thus incorrect predictions and uncertainty underestimation. Wider uncertainty bounds in output space, as seen in figure 5, reflect more adequately variations in pulmonary pressure due to the natural inter-subject factors (e.g. effects of the respiratory cycle). These are well known [6] and should be contained within the uncertainty bounds of the model.

To our knowledge, our study is one of the first to focus on parameter estimation in cardiovascular modelling while incorporating model mismatch. A notable exception is Lei *et al.* [19], who explore model discrepancy in cardiac electrophysiology, and the authors also show through synthetic studies that ignoring the model-form uncertainty produces biased predictions and uncertainty underestimation, which agrees with our findings.

### 7.2. Vessel wall model

The WAIC scores in table 4 suggest that the nonlinear wall model (model I) is better supported by the MPA-pressure data than the linear wall model (model G), since the former model registers a lower WAIC score. Our analysis indicates that, out of all the models investigated, the model that is most likely under the data is the nonlinear model with a slight dependence on the vessel radius, i.e. model I, recording the lowest WAIC score. This finding agrees with other studies—e.g. the study by Valdez *et al.* [44] on pressure area dynamics in systemic arteries of control sheep and the study by Pilhwa *et al.* [7] analysing distensibility of pulmonary arteries in control mice. The study in [45] provided experimental stress–strain relations in control and hypoxic pulmonary arteries, illustrating a predominant viscoelastic effect and further suggesting that a nonlinear elastic wall is more appropriate for modelling pulmonary haemodynamics. A visual comparison of the measured data with the predictions obtained using the linear or nonlinear model (figure 5) cannot be used as an objective metric to choose between models since the model which gives the closest prediction in Euclidean space to the measured data is not necessarily the most consistent with the data (e.g. for mis-specified mechanistic models). This emphasizes the need to carry out a formal model selection analysis.

### 7.3. Vessel wall stiffness

Out of all linear models, model G, i.e. the model with vessel-specific stiffness (with model mismatch) describes best the pressure data (table 4). As expected physiologically, an increased wall stiffness leads to increased systolic and pulse pressures (figure 7), with more dynamic changes in these values occurring at a lower stiffness range. Table 4 shows that the estimated stiffness in the linear models are within this range, suggesting accurate depiction of healthy haemodynamics, regardless of stiffness model. Additionally, given the data, an exponential radius dependence of the stiffness (model E) leads to non-influential parameters with nearly flat posteriors (figures 6 and 10).

Regarding the nonlinear models, our model selection results support a slight radius dependent stiffness—model I. Previous investigations [46] have shown that both wall thickness (*h*) and tissue properties (*E*) are drastically different in pulmonary arteries in pulmonary hypertension. This encourages future investigations into whether our model selection results are consistent in specimens with pulmonary hypertension.

### 7.4. Model fits

The pressure predictions shown in figure 8 deviate from model to model in arteries distal to the left and right pulmonary artery (vessels 2 and 3, respectively). While predictions look qualitatively similar, it is clear that the model used can lead to significant changes in downstream predictions. An understanding of how model type affects predictions down the pulmonary arteries is critical for future use of mathematical models in disease prognostication. For instance, pulmonary diseases like pulmonary hypertension remodel smaller arteriolar segments initially, making vessel stiffness a critical parameter in the development of disease [47–49]. The flow and pressure–area graphs show a more dramatic change between model types, which is expected as distal flow and dynamic area data are not available. This variability is important when considering lesions, i.e. pulmonary emboli, that can lead to obstructions in the pulmonary arteries, limiting perfusion to the alveoli for blood re-oxygenation. The pressure–area relations in figure 9 show that the inferred parameters for the nonlinear wall-model provide a nearly linear pressure–area curve, contrary to the findings in [45]. We suspect that inference using both pressure and dynamic area data will better illustrate dissimilarities between the two wall models, and hypothesize that additional pressure–flow data in distal arteries would allow for inference of additional Windkessel scaling factors in the network.

### 7.5. Parameter unidentifiability

Our analysis of the linear model with exponential radius-dependent stiffness shows that using complementary data from downstream vessels does not resolve the unidentifiability of *f*_1_ and *f*_2_ in equation (3.5) (figure 10). Thus, additional pressure data do not carry information about the non-influential parameters. If the model has structural unidentifiabilities, subsequent predictions are unreliable, and can lead to spurious diagnoses or sub-optimal treatments [50,51]. For this reason, it is imperative that in our exponential radius-dependent models, the entire expression *χ*(*f*_1_, *f*_2_, *f*_3_) in equation (3.5) is interpreted, and not the individual parameters, *f*_1_, *f*_2_, *f*_3_.

### 7.6. Future experimental design

Our analysis reveals that when complementary data are used, the parameters *f*_3_, *ψ*_1_, *ψ*_2_ and *c* are more accurately estimated (but not *f*_1_ and *f*_2_; see §7.5 for a justification), and our study allows the reduction in the estimation uncertainty to be quantified (table 5). This may be used in future experimental design, when deciding whether to record measurements in vessels beyond MPA. Furthermore, results in figure 10 show that the true parameter values are accurately inferred, validating our inference procedure.

### 7.7. Real-time treatment planning

A long-term goal of our project is real-time, personalized treatment planning. Therefore, once the model selection procedure finds the ‘best’ model, predictions from that model should be computationally efficient. We show that this can be accomplished using efficient surrogate models in place of the computationally expensive PDE model (see table 2; electronic supplementary material, S9). In principle, the emulation approach could be performed for the vessel-specific nonlinear model, which was not explored in this study due to very computationally costly simulations. However, emulation would most likely require high efforts due to a large number of model parameters (i.e. 42 vessel-stiffness parameters, three Windkessel factors and two error parameters). These parameters might be unidentifiable, leading to a high-dimensional parameter domain needing coverage by the emulator, requiring a long time for training, as well as the implementation of more sophisticated GPs (e.g. sparse GPs [52]). This is certainly a highly topical problem with formidable methodological challenges for cutting edge machine learning research. However, pursuing this is far beyond the remit of the present work.

## 8. Limitations and future directions

The physiological conclusions from the model selection analysis according to which the nonlinear model is preferred over the linear model is based on the study of just one mouse. Moreover, it would be interesting to compare the performance of the asymptotically based WAIC approach for model selection to an approach which does not rely on asymptotics, e.g. marginal likelihood [53], whose calculation, however, comes at a significantly higher computational cost.

Given that this is a retrospective data analysis study, with limited data (a single MPA pressure signal), unspecified machine precision for data measurement, unknown data smoothing and averaging technique applied to the raw data, and lack of prior knowledge of the model discrepancy function, it is intrinsically impossible to distinguish and separately incorporate the model discrepancy and the measurement errors (noise) model. In principle, a strongly informative prior on the model discrepancy function could help separate the contributions from the model and measurement errors [54]. Multi-scale vessel wall models that include fluid–structure interactions at individual cell level, or 3D computational fluid-dynamics models, may be too complex for inference, but could refine prior knowledge. Running forward simulations with both high and medium fidelity models for a space filling design in parameter space and then fitting a GP to the differences in output space could give a more realistic prior for future inference applications. However, these higher fidelity fluid-dynamics models come with their own modelling assumptions and non-measurable parameters, hence a prior built on these models may be inherently biased.

This study analyses both boundary condition and vessel stiffness parameters. We only examined one boundary condition model and estimated scaling factors that adjust nominal Windkessel parameters in each terminal vessel. This boundary condition ignores the fractal structure of the downstream vascular, which is accounted for by the structured tree model [5,10,48]. However, these methods can be used with any boundary condition, and will be pursed in the future.

The uncertainty in the vessel network, which is kept fixed, will also be modelled in the future [3,11]. Minor losses at junctions, unaccounted for here, are also subject to uncertainty and can further introduce a model mismatch. This is systematically addressed by the proposed inference scheme, which provides a mechanism to prevent such model mismatch from causing any bias in the parameter estimation and haemodynamic predictions.

Additionally, the MPA inflow boundary condition could be replaced by a coupling of the MPA with a right ventricle model [55].

## 9. Conclusion

Our study uses Bayesian analysis techniques to approximately infer the posterior distribution with the aim to quantify the uncertainty of model parameters and haemodynamic predictions in a 1D fluid-dynamics model of the pulmonary circulation.

Our main contribution is to draw attention to an often neglected source of uncertainty: in the mathematical model form, caused by the discrepancy between the real system and the model, and in the measurements due to the wrong noise model (jointly called ‘model mismatch’).

Additionally, we explored several mathematical models (a linear and a nonlinear wall model with different vessel wall stiffness assumptions: constant, vessel-specific or radius-dependent stiffness), and error models (via the inclusion of a model mismatch). We implemented Bayesian model selection based on WAIC to find the model that can most accurately predict the MPA pressure and provide adequate uncertainty quantification in the pressure predictions.

Our study clearly demonstrates that the widely used least-squares fit method ignores model mismatch, biasing parameter estimates and model predictions, and underestimating uncertainty in parameter and output space. We circumvent these issues by incorporating the model mismatch using GPs.

Additionally, we found that the MPA-measured pressure data best supports the nonlinear wall model with a weak exponential radius-dependent stiffness.

Lastly, our synthetic study validates our inference procedure, identifies those parameters that benefit from complementary data distal to the MPA, and quantifies the reduction in their intrinsic estimation uncertainty, which may help better design future experiments.

## Supplementary Material

Supplementary material

## Supplementary Material

Pulmonary blood pressure data

## Supplementary Material

Pulmonary blood flow data

## Endnotes

1 http://vmtk.org

2 For a comparison, the elapsed time required to complete simulations with the linear model G was roughly six weeks.

## Data accessibility

This article has no additional data.
