# Supplementary material for: Assessing model mismatch and model selection in a Bayesian uncertainty quantification analysis of a fluid-dynamics model of pulmonary blood circulation
Source: J R Soc Interface. 2020 Dec 23;17(173):20200886. doi: 10.1098/rsif.2020.0886 (PMC7811590; doi:10.1098/rsif.2020.0886)
Supplement: Supplementary material [file rsif20200886supp1.pdf]

# Supplementary Material for 'Assessing model mismatch and model selection in a Bayesian uncertainty quantification analysis of a fluid-dynamics model of pulmonary blood circulation'

L. Mihaela Paun[1], Mitchel J. Colebank[2], Mette S. Olufsen[2], Nicholas A. Hill[1] and Dirk Husmeier[1]

*1) School of Mathematics and Statistics, University of Glasgow, Glasgow G12 8SQ, UK*
*2) Department of Mathematics North Carolina State University, Raleigh, NC, 27695, USA*

## 1. Relevant statistical methodology

### 1.1. Bayesian Model Selection: WAIC

Should we be interested in carrying out model selection, several selection criteria are available: Akaike Information Criterion (AIC) [1], Bayesian Information Criterion (BIC) [2], Deviance Information Criteria (DIC) [3], the marginal likelihood [4] or Watanabe Akaike Information Criterion (WAIC) [5]. AIC and BIC have an asymptotic foundation, but exhibit a poor small-sample behaviour, DIC is not suitable for singular or multimodal likelihoods [5], while the marginal likelihood [4] entails high computational complexity when numerically stable procedures are used, such as thermodynamic integration [6, 7], and numerical instability of computationally affordable schemes like the harmonic mean estimator [8]. WAIC is used in this study as it is an asymptotic method with better small-sample behaviour than AIC, and its computation is straightforward once the MCMC posterior samples are obtained. WAIC [5] is calculated using the whole posterior distribution – see eq. (1). It takes the log posterior predictive density (first term) and adjusts for overfitting by adding a correction for the effective number of parameters (second term) [9],

$$\text{WAIC} = -2\sum_{i=1}^{n}\log\int p(y_i|\boldsymbol{\theta})p(\boldsymbol{\theta}|\mathbf{y})d\boldsymbol{\theta} + 2\sum_{i=1}^{n}\text{var}_{\boldsymbol{\theta}|\mathbf{y}}(\log p(y_i|\boldsymbol{\theta})), \quad (1)$$

which in practice is computed as follows:

$$\text{computed WAIC } = -2\sum_{i=1}^{n}\log\left(\frac{1}{S}\sum_{s=1}^{S}p(y_i|\boldsymbol{\theta}^s)\right) + 2\sum_{i=1}^{n}V_{s=1}^{S}(\log p(y_i|\boldsymbol{\theta}^s)), \quad (2)$$

where $\boldsymbol{\theta}^s, s = 1, ...S$, are posterior samples from $p(\boldsymbol{\theta}|\mathbf{y})$, and the following holds: $V_{s=1}^{S}a_s = \frac{1}{S-1}\sum_{s=1}^{S}(a_s - \bar{a})^2$.

### 1.2. Gaussian Processes for Regression

#### 1.2.1. Definition

A stochastic process $f = f(\mathbf{t})_{\mathbf{t}\in\mathcal{T}}$ is defined as a Gaussian process (GP) if the random variables $\mathbf{f} = (f(\mathbf{t}_1), \ldots f(\mathbf{t}_n))$ are jointly normal for any inputs $\mathbf{t}_i \in \mathcal{R}^{d\times 1}$, with $i = 1, \ldots n$: $\mathbf{f} \sim \mathcal{MVN}(\mathbf{m}, \mathbf{K})$, where

*L. Mihaela Paun
*Email address:* l.paun.1@research.gla.ac.uk (L. Mihaela Paun[1], Mitchel J. Colebank[2], Mette S. Olufsen[2], Nicholas A. Hill[1] and Dirk Husmeier[1])

$\mathbf{m} = (m(\mathbf{t}_1), \ldots, m(\mathbf{t}_n))$ is the mean $n$-vector and $\mathbf{K} = [k(\mathbf{t}_i, \mathbf{t}_j)]_{i,j=1}^n$ is the $n \times n$ variance-covariance matrix of $\mathbf{f}$. In GP models [10, 11], inputs $\mathbf{T} = \{\mathbf{t}_1, \ldots \mathbf{t}_n\}$ are mapped into outputs by means of latent noiseless functions $\mathbf{f}$. A GP prior is placed on the distribution of these functions as a way to account for the uncertainty in the functional form,

$$\mathbf{f}(\mathbf{T})|\boldsymbol{\eta} \sim \mathcal{GP}(\mathbf{m}(\mathbf{T}), \mathbf{K}|\boldsymbol{\eta}), \tag{3}$$

where $\boldsymbol{\eta}$ contains the covariance function (kernel) hyperparameters. Typically, the mean of the GP is set to zero ($\mathbf{m}(\mathbf{T}) = \mathbf{0}$), i.e. the data are standardised to zero mean, which will henceforth be assumed. The covariance function, $k(\mathbf{t}, \mathbf{t}'|\boldsymbol{\eta})$, gives the smoothness and variability of the latent functions.

*1.2.2. Covariance functions (kernels)*

Several covariance functions are available, and they can be stationary, like the squared exponential kernel (most popular), the Matérn class kernel, the periodic kernel, or non-stationary, like the neural network kernel (see Chapter 4 in [10] for more details). For example, the squared exponential kernel has the form

$$k(\mathbf{t}, \mathbf{t}^{'}|\boldsymbol{\eta}) = \sigma_m^2 \exp\left(-\frac{||\mathbf{t} - \mathbf{t}^{'}||^2}{2l^2}\right), \tag{4}$$

where $\boldsymbol{\eta} = (\sigma_m^2, l)$, with $\sigma_m^2$ being the marginal variance of the function or response variable, equivalent to the signal variance, and $l$ being the lengthscale, which controls the flexibility of the function on the x-axis (the explanatory or input variable). Additionally, the neural network kernel has the form

$$k(\mathbf{t}, \mathbf{t}^{'}|\boldsymbol{\eta}) = \frac{2}{\pi} \arcsin\left(\frac{2\hat{\mathbf{t}}^\mathsf{T}\boldsymbol{\Sigma}\hat{\mathbf{t}}^{'}}{\sqrt{(1 + 2\hat{\mathbf{t}}^\mathsf{T}\boldsymbol{\Sigma}\hat{\mathbf{t}})(1 + 2\hat{\mathbf{t}}^{'\mathsf{T}}\boldsymbol{\Sigma}\hat{\mathbf{t}}^{'})}}\right), \tag{5}$$

where $\hat{\mathbf{t}} = (1 \quad \mathbf{t})^\mathsf{T}$ is an augmented vector ($\mathsf{T}$ superscript denotes the transposition of the vector), and $\boldsymbol{\Sigma}$ is a diagonal matrix containing the kernel hyperparameters, $\boldsymbol{\Sigma} = \mathrm{diag}(b, w)$, and $\boldsymbol{\eta} = (b, w)$ with $b$ controlling the amount of offset of the functions from the origin and $w$ controlling the scaling on the x-axis.

*1.2.3. Prediction*

Using GPs we can also perform prediction at unseen input values. The predictive distribution of a new latent function, $\tilde{f} = f(\tilde{t})$, is also Gaussian [10], as follows:

$$\tilde{f}|D, \boldsymbol{\eta}, \sigma_n^2 \sim \mathcal{N}(m_\mathrm{p}(\tilde{\mathbf{t}}), k_\mathrm{p}(\tilde{\mathbf{t}}, \tilde{\mathbf{t}}'|\boldsymbol{\eta})), \tag{6}$$

$$m_\mathrm{p}(\tilde{\mathbf{t}}) = \boldsymbol{k}(\tilde{\mathbf{t}}, \mathbf{T}|\boldsymbol{\eta})(\mathbf{K} + \sigma_n^2\mathbf{I})^{-1}\boldsymbol{\Gamma}, \tag{7}$$

$$k_\mathrm{p}(\tilde{\mathbf{t}}, \tilde{\mathbf{t}}'|\boldsymbol{\eta}) = k(\tilde{\mathbf{t}}, \tilde{\mathbf{t}}'|\boldsymbol{\eta}) - \boldsymbol{k}(\tilde{\mathbf{t}}, \mathbf{T}|\boldsymbol{\eta})(\mathbf{K} + \sigma_n^2\mathbf{I})^{-1}\boldsymbol{k}(\mathbf{T}, \tilde{\mathbf{t}}'|\boldsymbol{\eta}), \tag{8}$$

where $D = \{\mathbf{t}, \boldsymbol{\Gamma}\}$ is the set of training points, $\boldsymbol{k}(\tilde{\mathbf{t}}, \mathbf{T}|\boldsymbol{\eta})$ is a vector valued kernel function, $\boldsymbol{k}(\tilde{\mathbf{t}}, \mathbf{T}) : \mathcal{R}^{d\times 1} \times \mathcal{R}^{d\times n} \to \mathcal{R}_+^{1\times n}$, and $\boldsymbol{\eta}$ and $\sigma_n^2$ have been obtained based on the training data $D$.

*1.3. Gaussian Processes for Classification*

Besides recording a real-valued dependent variable, we may record a binary variable $\boldsymbol{\lambda} = (\lambda_1, \ldots \lambda_n)$ with $\lambda_i \in \{0, 1\}$ associated with inputs $\mathbf{T} = \{\mathbf{t}_i\}_{i=1}^n$, where 0: failure and 1: success. GPs can also be applied to classification problems, where we are for example interested in deciding on the success of the PDE evaluation for a particular parameter configuration (i.e. the fluid-dynamics model makes physical assumptions that for certain parameter values are violated, which may leave the PDEs without solutions). To do so, we can build a GP classification model that places a GP prior over the distribution of noiseless latent functions, $\mathbf{f}$, as follows:

$$\begin{aligned} \lambda_i|f(\mathbf{t}_i) &\sim \mathrm{Bernoulli}(p(\lambda_i = 1|f(\mathbf{t}_i))), \\ \mathbf{f}(\mathbf{T})|\boldsymbol{\eta} &\sim \mathcal{GP}(\mathbf{m}(\mathbf{T}), \mathbf{K}|\boldsymbol{\eta}), \\ \boldsymbol{\eta} &\sim p(\boldsymbol{\eta}), \end{aligned} \tag{9}$$

where $\boldsymbol{\eta}$ contains the kernel hyperparameters.

The binary observations (class labels), $\boldsymbol{\lambda}$, are drawn from a Bernoulli distribution with a success probability $p(\lambda_i = 1|f(\mathbf{t}_i))$, and likelihood shown in eq. (11). The success probability is related to the function $f(\mathbf{t}_i)$ via the sigmoid function, $\text{sig}(f(\mathbf{t}_i)) = (1 + \exp(-f(\mathbf{t}_i)))^{-1}$, which transforms the probability into the unit interval, [0,1], as shown in eq. (10):

$$p(\lambda_i = 1|f(\mathbf{t}_i)) = \text{sig}(f(\mathbf{t}_i)); \quad p(\lambda_i = 0|f(\mathbf{t}_i)) = 1 - p(\lambda_i = 1|f(\mathbf{t}_i)), \tag{10}$$

$$\text{Likelihood} : p(\lambda_i|f(\mathbf{t}_i)) = (\text{sig}(f(\mathbf{t}_i)))^{\lambda_i}(1 - \text{ sig}(f(\mathbf{t}_i)))^{(1-\lambda_i)} \text{ for a binary outcome } \lambda_i \in \{0, 1\}. \tag{11}$$

The latent functions, $\mathbf{f}$, can be integrated out from the conditional posterior distribution given the hyperparameters $\boldsymbol{\eta}$, to obtain the marginal likelihood (eq. (12)), which has no closed-form solution:

$$p(\boldsymbol{\lambda}|\mathbf{T}, \boldsymbol{\eta}) = \int p(\boldsymbol{\lambda}, \mathbf{f}|\mathbf{T}, \boldsymbol{\eta})d\mathbf{f} = \int p(\boldsymbol{\lambda}|\mathbf{f})p(\mathbf{f}|\mathbf{T}, \boldsymbol{\eta})d\mathbf{f}. \tag{12}$$

With a non-Gaussian likelihood, we can run MCMC [12], or approximate the conditional posterior distribution by a Gaussian form using the Laplace approximation [13], variational inference [14] or expectation propagation (EP) [15].

## 2. GPs for model mismatch in Section 4.2 of the main paper

For the model mismatch problem discussed in our study, we follow Kennedy et al. [16], and place a GP prior on the model mismatch term $\boldsymbol{\Gamma}$, expressed as

$$\begin{aligned} \mathbf{y}(\mathbf{t}) &= \mathbf{m}(\boldsymbol{\theta}, \mathbf{t}) + \boldsymbol{\Gamma}(\mathbf{t}), \\ \boldsymbol{\Gamma}(\mathbf{t}) &= \boldsymbol{\zeta}(\mathbf{t}) + \boldsymbol{\epsilon}(\mathbf{t}), \end{aligned} \tag{13}$$

where $\mathbf{y}(\mathbf{t})$ is the measured pressure data at time points collected in $\mathbf{t}$ (which is an $n \times 1$ time series), $\mathbf{m}(\boldsymbol{\theta}, \mathbf{t})$ is the simulator output (i.e. the data prediction from the mathematical model evaluated at the parameters $\boldsymbol{\theta}$), $\boldsymbol{\zeta}(\mathbf{t})$ is the model discrepancy function representing the discrepancy between the real system and the prediction from the mathematical model. Also, $\boldsymbol{\epsilon}(\mathbf{t})$ is the noise model function representing the measurement errors, which due to the nature of the data, are longitudinally correlated in time, and $\boldsymbol{\Gamma}(\mathbf{t})$ is the model mismatch, represented by the residuals. Note that in the present article, we distinguish between *model discrepancy* and *model mismatch* in the way described above, so these words are not used synonymously.

While Kennedy's formulation of eq (13) allows for model discrepancy (modelled with a GP), it makes the assumption of iid measurement errors, i.e. assumes that $\boldsymbol{\epsilon} \sim \mathcal{MVN}(\mathbf{0}, \sigma_n^2\mathbf{I})$. In our formulation we generalise this by allowing for correlated measurement errors.

To help with the GP set-up for the model mismatch (i.e. for the residuals), we further express the model mismatch in terms of latent functions $\mathbf{f}$ as

$$\begin{aligned} \boldsymbol{\Gamma}(\mathbf{t}) &= \mathbf{f}(\mathbf{t}) + \mathbf{u}(\mathbf{t}), \\ \mathbf{u}(\mathbf{t}) &\sim \mathcal{MVN}(\mathbf{0}, \sigma_n^2\mathbf{I}), \end{aligned} \tag{14}$$

where $\sigma_n^2$ is the noise variance of the residuals, assumed iid Gaussian.

Next, we place a GP prior on the latent functions $\mathbf{f}$ and following eq (3), we write

$$\mathbf{f}(\mathbf{t})|\boldsymbol{\eta} \sim \mathcal{GP}(\mathbf{0}, \mathbf{K}|\boldsymbol{\eta}). \tag{15}$$

This GP captures the correlation stemming from both the model discrepancy $\boldsymbol{\zeta}(\mathbf{t})$ and the measurement errors $\boldsymbol{\epsilon}(\mathbf{t})$, defined in eq (13). In principle we could place a GP prior on the model discrepancy function $\boldsymbol{\zeta}$, i.e. $\boldsymbol{\zeta}(t) \sim \mathcal{GP}(\mathbf{0}, \mathbf{K_1}|\boldsymbol{\eta_1})$, and another GP prior on the noise model function, i.e. $\boldsymbol{\epsilon}(\mathbf{t}) \sim \mathcal{GP}(\mathbf{0}, \mathbf{K_2}|\boldsymbol{\eta_2})$, but since this is a retrospective data analysis study, it is intrinsically impossible to distinguish and separately model these two terms; instead we place a GP on the sum of the two terms, and in eq (15), $\mathbf{K} = \mathbf{K_1} + \mathbf{K_2}$, assuming that the correlation between the model mismatch processes (i.e. model discrepancy and measurement errors) is zero.

## 3. Using GPs to obtain the pressure data likelihood

As outlined in Section 4.2 of the main paper, we use GPs to estimate the covariance matrix of the residuals (and implicitly allow for model mismatch), which will be subsequently used in the calculation of the pressure data likelihood.

As explained in Section 4.1 of the main paper, when Gaussian correlated errors are assumed, $\mathbf{y}(\mathbf{t}) \sim \mathcal{MVN}(\mathbf{m}(\boldsymbol{\theta}, \mathbf{t}), \mathbf{C})$,

$$p(\mathbf{y}|\boldsymbol{\theta}, \mathbf{C}) = \det(2\pi\mathbf{C})^{-\frac{1}{2}} \exp\left(-\frac{1}{2}(\mathbf{y}(\mathbf{t}) - \mathbf{m}(\boldsymbol{\theta}, \mathbf{t}))^{\mathrm{T}}\mathbf{C}^{-1}(\mathbf{y}(\mathbf{t}) - \mathbf{m}(\boldsymbol{\theta}, \mathbf{t}))\right). \quad (16)$$

$\mathbf{C}$ in eq (16) is the covariance matrix of the residuals, i.e. $\mathbf{C} = \mathbf{K} + \sigma_n^2\mathbf{I}$, where $\mathbf{K}$ is given in eq (15) and $\sigma_n^2$ is given in eq (14).

In addition, eq (14) allows to obtain the limiting case of iid errors, i.e. no model mismatch, by constraining the kernel hyperparameters. For example, for the neural network covariance function used in this study (eq (5)), setting $w = 0$ and $b = 0$ allows the covariance matrix $\mathbf{K}$ become a zero matrix, and $\sigma^2 = \sigma_n^2$.

## 4. Checking for Gaussianity of the errors

An analysis of the residuals allows to check if they are consistent with the assumption of a Gaussian distribution, which we have made in the analysis. To test this, we have constructed a probability-probability (PP) plot comparing the distribution of the residuals to the normal distribution. A PP plot finds the quantiles of the residuals and of the Gaussian (theoretical) distribution, and the quantiles are converted into probability values (cumulative distribution functions, cdfs); the cdfs of the two data sets are plotted against each other. If the distribution of the residuals is indeed Gaussian, this probability plot should show points lying on a 45° line. Note that a PP plot is similar to a QQ plot, which shows the quantiles of the data sets against each other, however a PP plot is more easily interpretable.

We have carried out this analysis for all models listed in Tables 1 and 2 of the main paper. For those models where we ignore the model mismatch (Models A, D and F), the residuals are computed from equation 13 in the main paper. We sample the parameters of the PDEs ($\boldsymbol{\theta}$) from the posterior distribution, subtract each of the resulting PDE solutions from the data, and use the residuals to obtain the probability plot. For those models where we allow for model mismatch (all other models shown in Tables 1 and 2 of the main paper), the residuals are computed from equation 14 in the main paper. We sample both the parameters of the PDEs $\boldsymbol{\theta}$ and the Gaussian process realisations of the model mismatch term $\mathbf{f}(\mathbf{t})$ from the posterior distribution. For each sample, we subtract the PDE solution plus the Gaussian process realisation from the data, and again proceed to obtain the probability plot for the residuals.

Our results are shown in Figure 1 below. When not allowing for model mismatch, the residuals show a certain deviation from the assumed normal distribution, in that the probability plots deviate from the 45° line in the tails of the distribution. To get a better feel for how strong this deviation is, we have obtained the probability plots for a series of t-distributions with varying degrees of freedom (a Gaussian distribution is a t-distribution with an infinite number of degrees of freedom). We kept the one that led to a probability plot that is visually similar to the one obtained for our residuals. This distribution is plotted in Figure 2 below. It can be seen that the deviation from the Gaussian distribution is rather modest, with slightly heavier tails. When allowing for model mismatch, the probability plots show excellent agreement with the 45° line, which confirms that the assumption of a normal distribution is justified.

In summary: If we do not allow for model mismatch, the Gaussian assumption for the residuals is slightly violated, in that our probability plots suggest that the actual distribution has slightly heavier tails. When we explicitly correct for model mismatch, our Gaussian assumption for the residuals is confirmed by the probability plots. Hence, although the model mismatch approach proposed in our paper has been designed with the objective to correct for error correlation, it is interesting to notice that it also achieves better agreement with the assumed Gaussian distribution for the residuals.

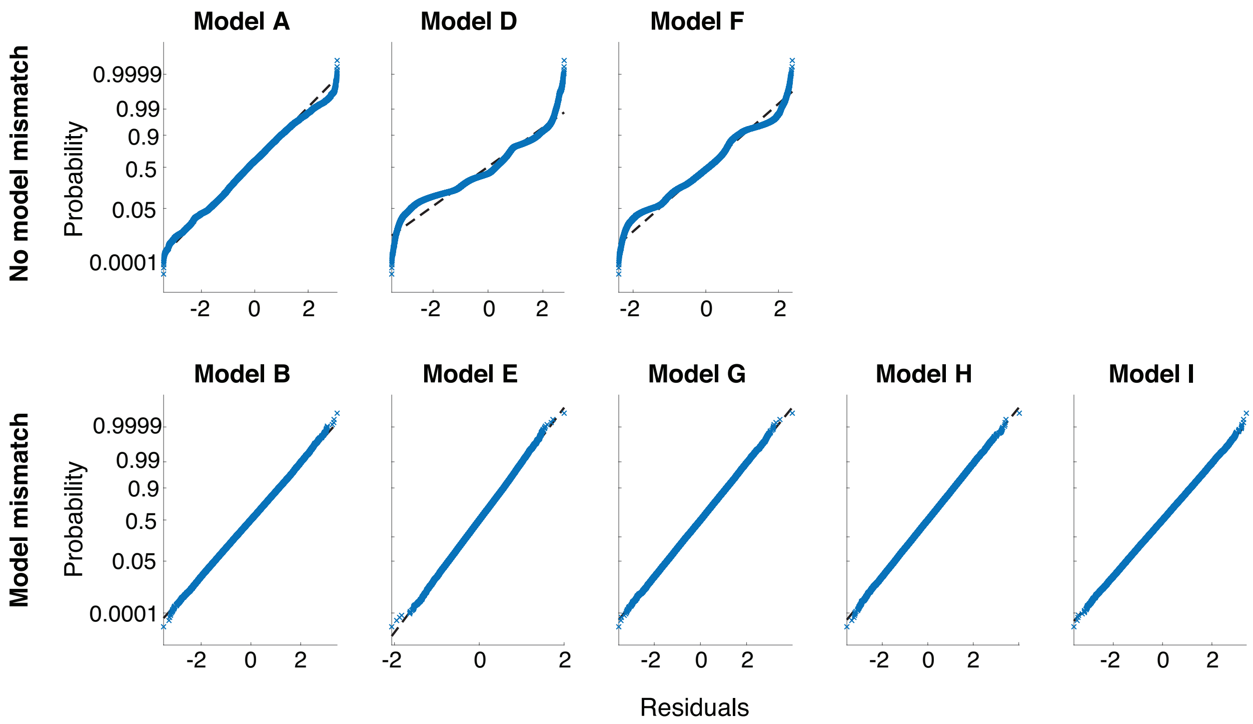


Figure 1: Checking for Gaussianity of the errors for all models in Tables 1 and 2 of the main paper by constructing and displaying probability-probability (PP) plots for the distribution of the errors against the normal distribution (first row: models neglecting model mismatch, second row: models allowing for model mismatch).

## 5. Details of the hierarchical Bayesian model

This section compliments Section 4.3.1 in the main paper.

### *5.1. General principles of our hierarchical Bayesian model*

When employing the hierarchical model, apriori, we assume that all parameters are conditionally independent given $m_\chi$ and $\sigma_\chi^2$, but marginally (after integrating out $m_\chi, \sigma_\chi^2$) dependent. We also assume that the parameters come from a "population" Normal distribution, $\mathcal{N}(m_\chi, \sigma_\chi^2)$, and $m_\chi \sim \mathcal{N}(m^*, \sigma^{2*})$ and $\sigma_\chi^2 \sim \mathcal{IG}(\alpha^*, \beta^*)$ (where $\mathcal{IG}$ is the inverse gamma distribution, which is a conjugate prior). The hyper-hyperparameters $m^*, \sigma^{2*}, \alpha^*, \beta^*$ take fixed values and are set such that there is roughly 90% prior probability that the stiffness parameters are within the physiologically plausible range (details in Section 8.3 of the Supplement). Since $m_\chi$ and $\sigma_\chi^2$ are not fixed, the random variables $\chi_1, \dots \chi_k$ are not d-separated (see Section 10.5.1 in [17]), which enables information coupling. The choice of the priors is motivated by conjugacy, and leads to closed-form posterior distributions (more details in Section 6 of the Supplement).

This model assumes exchangeability apriori (see Section 5.2 in [18]), i.e. parameters $(\chi_1, \dots \chi_k)$ are exchangeable in their joint distribution, which means that swapping the $i^{\text{th}}$ and $j^{\text{th}}$ vessel stiffness leads to the same joint distribution. This is a limitation of the current work, as vessel stiffness may depend on the vessel radius. However, we argue that the independence of the stiffness from the radius is only assumed apriori, and is most likely overruled aposteriori if a radius dependence exists.

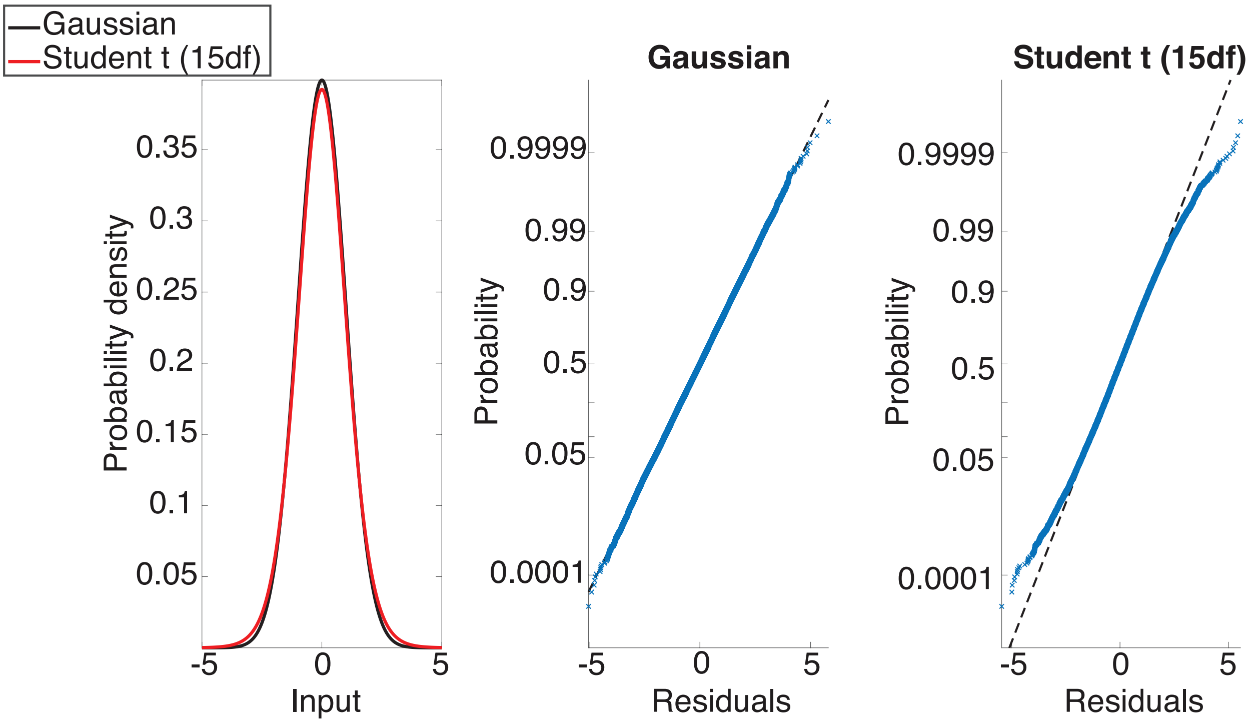


Figure 2: Comparing a Gaussian distribution to a Student t-distribution with 15 degrees of freedom.

*5.2. Mathematical details of our hierarchical Bayesian model*

*5.2.1. Standard Gibbs sampler*

In a hierarchical Bayesian model, the probability of a selected parameter conditional on all the other parameters is equal to the probability of the parameter conditional on its Markov blanket:

$$p(m_\chi|\text{everything else}) \;=\; p(m_\chi|\text{Markov blanket of } m_\chi).$$

The Markov blanket is the set of parents, children and co-parents. For $m_\chi$, the parents are $m^*, \sigma^{2*}$, the children are $\boldsymbol{\chi} = (\chi_1, \ldots, \chi_k)$, and there is one co-parent: $\sigma_\chi^2$. This gives:

$$p(m_\chi|\text{everything else}) \;=\; p(m_\chi|m^*, \sigma^{2*}, \chi_1, \ldots, \chi_k, \sigma_\chi^2).$$

The conditional probability is proportional to the joint probability:

$$p(m_\chi|m^*, \sigma^{2*}, \chi_1, \ldots, \chi_k, \sigma_\chi^2) \;\propto\; p(m_\chi, m^*, \sigma^{2*}, \chi_1, \ldots, \chi_k, \sigma_\chi^2).$$

Next, we factorize the joint probability according to the factorization rules for directed graphical models, applied to Figure 2 of the main paper:

$$p(m_\chi, m^*, \sigma^{2*}, \chi_1, \ldots, \chi_k, \sigma_\chi^2) \;=\; p(m_\chi|m^*, \sigma^{2*}) \prod_{i=1}^{k} p(\chi_i|m_\chi, \sigma_\chi^2).$$

According to Figure 2 (main paper), all distributions on the right-hand side are Gaussian, so we get:

$$p(m_\chi|\text{everything else}) \;\propto\; \mathcal{N}(m_\chi|m^*, \sigma^{2*}) \prod_{i=1}^{k} \mathcal{N}(\chi_i|m_\chi, \sigma_\chi^2).$$

Now, we write out the expressions for the Gaussian, and keep all the terms that explicitly depend on $m_\chi$. The other terms are constant with respect to $m_\chi$ and get absorbed in the normalization constant. This leads to:

$$\begin{aligned}
p(m_\chi|\text{everything else}) &\propto \mathcal{N}(m_\chi|m^*, \sigma^{2*}) \prod_{i=1}^{k} \mathcal{N}(\chi_i|m_\chi, \sigma_\chi^2) \\
&= \exp\left(\frac{-1}{2\sigma^{2*}}(m_\chi - m^*)^2 + \frac{-1}{2\sigma_\chi^2}\sum_{i=1}^{k}(\chi_i - m_\chi)^2\right) \\
&\propto \exp\left(\frac{-1}{2} m_\chi^2 \left[\frac{k}{\sigma_\chi^2} + \frac{1}{\sigma^{2*}}\right] + m_\chi \left[\frac{m^*}{\sigma^{2*}} + \frac{1}{\sigma_\chi^2}\sum_{i=1}^{k}\chi_i\right]\right).
\end{aligned}$$

Completing the square and normalizing this distribution yields:

$$p(m_\chi|\text{everything else}) = \mathcal{N}\left(m_\chi \middle| \frac{\frac{m^*}{\sigma^{2*}} + \frac{1}{\sigma_\chi^2}\sum_{i=1}^{k}\chi_i}{\frac{1}{\sigma^{2*}} + \frac{k}{\sigma_\chi^2}}, \left[\frac{1}{\sigma^{2*}} + \frac{k}{\sigma_\chi^2}\right]^{-1}\right). \tag{17}$$

In words: the mean of the conditional posterior distribution is a weighted sum of $m^*, \chi_1, \ldots, \chi_k$, weighted by their respective precision. The precision of the conditional posterior distribution is a sum of the individual precisions of $m^*, \chi_1, \ldots, \chi_k$. This makes intuitively sense: each of the variables in $\{m^*, \chi_1, \ldots, \chi_k\}$ contributes a piece of information whose value is its precision. The overall precision gets higher as you pass information from more variables to $m^*$ (i.e. its inverse, the variance, gets reduced).

For $\sigma_\chi^2$, we proceed in the same way:

$$p(\sigma_\chi^2|\text{everything else}) = p(\sigma_\chi^2|\text{Markov blanket of } \sigma_\chi^2).$$

Again, the Markov blanket is the set of parents, children and co-parents. For $\sigma_\chi^2$, the parents are $\alpha^*, \beta^*$, the children are $\boldsymbol{\chi} = (\chi_1, \ldots, \chi_k)$, and there is one co-parent: $m_\chi$. So we get:

$$p(\sigma_\chi^2|\text{everything else}) = p(\sigma_\chi^2|\alpha^*, \beta^*, \chi_1, \ldots, \chi_k, m_\chi).$$

The conditional probability is proportional to the joint probability:

$$p(\sigma_\chi^2|\text{everything else}) \propto p(\sigma_\chi^2, \alpha^*, \beta^*, \chi_1, \ldots, \chi_k, m_\chi).$$

Now we factorize the joint probability according to the factorization rules for directed graphical models, applied to Figure 2 of the main paper. Again, we only need to keep those terms that explicitly depend on $\sigma_\chi^2$, as the other terms get absorbed in the normalization constant. Since our prior is conjugate, we get a distribution in closed form (an inverse gamma distribution), as follows:

$$\begin{aligned}
p(\sigma_\chi^2|\text{everything else}) &\propto \mathcal{IG}(\alpha^*, \beta^*) \prod_{i=1}^{k} \mathcal{N}(\chi_i|m_\chi, \sigma_\chi^2) \\
&\propto \left[(\sigma_\chi^2)^{-\alpha^*-1} \exp\left(-\frac{\beta^*}{\sigma_\chi^2}\right)\right]\left[\left(\frac{1}{\sigma_\chi^2}\right)^{\frac{k}{2}} \exp\left(\frac{-1}{2\sigma_\chi^2}\sum_{i=1}^{k}(\chi_i - m_\chi)^2\right)\right] \\
&\propto \left[(\sigma_\chi^2)^{-\left(\alpha^*+\frac{k}{2}\right)-1} \exp\left[-\frac{1}{\sigma_\chi^2}\left(\beta^* + 0.5\sum_{i=1}^{k}(\chi_i - m_\chi)^2\right)\right]\right].
\end{aligned}$$

Normalizing this distribution yields:

$$p(\sigma_\chi^2|\text{everything else}) \;=\; \mathcal{IG}\left(\alpha^* + \frac{k}{2}, \beta^* + 0.5\sum_{i=1}^{k}(\chi_i - m_\chi)^2\right). \tag{18}$$

So we now have a Gibbs sampler that can be used for sampling from the posterior distribution, iteratively sampling from

- $p(m_\chi|m^*, \sigma^{2*}, \chi_1, \ldots, \chi_k, \sigma_\chi^2)$ in eq (17),
- $p(\sigma_\chi^2|\alpha^*, \beta^*, \chi_1, \ldots, \chi_k, m_\chi)$ in eq (18),
- $p(\boldsymbol{\chi}, r_1, r_2, c|m_\chi, \sigma_\chi^2, \mathbf{y}, \alpha, \beta)$,

where the sampling of $\boldsymbol{\chi} = (\chi_1, \ldots, \chi_k)$ and $r_1, r_2$ and $c$ cannot be done analytically and follows a Metropolis-Hastings within Gibbs scheme.

*5.2.2. A first attempt at collapsing*

We will marginalise over $m_\chi$.

$$\begin{aligned}
p(m_\chi, \sigma_\chi^2|\text{everything else}) \;&\propto\; \mathcal{IG}(\sigma_\chi^2|\alpha^*, \beta^*)\mathcal{N}(m_\chi|m^*, \sigma^{2*})\prod_{i=1}^{k}\mathcal{N}(\chi_i|m_\chi, \sigma_\chi^2) \\
&\propto\; \left(\frac{1}{\sigma_\chi^2}\right)^{\alpha^*+1} \exp\left(\frac{-\beta^*}{\sigma_\chi^2}\right)\left(\frac{1}{\sigma_\chi^2}\right)^{k/2} \\
&\quad\; \exp\left(\frac{-1}{2\sigma^{2*}}(m_\chi - m^*)^2 + \frac{-1}{2\sigma_\chi^2}\sum_{i=1}^{k}(\chi_i - m_\chi)^2\right) \\
&=\; \left(\frac{1}{\sigma_\chi^2}\right)^{\alpha^*+1}\left(\frac{1}{\sigma_\chi^2}\right)^{k/2} \exp\left(\frac{-\beta^*}{\sigma_\chi^2}\right)\exp\left(\frac{-1}{2\sigma_\chi^2}\sum_{i=1}^{k}(\chi_i - \overline{\chi})^2\right) \\
&\quad\; \exp\left(\frac{-1}{2\sigma^{2*}}(m_\chi - m^*)^2 + \frac{-k}{2\sigma_\chi^2}(\overline{\chi} - m_\chi)^2\right) \\
&\propto\; \left(\frac{1}{\sigma_\chi^2}\right)^{\alpha^*+1}\left(\frac{1}{\sigma_\chi^2}\right)^{(k-1)/2} \exp\left(\frac{-\beta^*}{\sigma_\chi^2}\right)\exp\left(\frac{-1}{2\sigma_\chi^2}\sum_{i=1}^{k}(\chi_i - \overline{\chi})^2\right) \\
&\quad\; \mathcal{N}(m_\chi|m^*, \sigma^{2*})\mathcal{N}\left(\overline{\chi}|m_\chi, \frac{\sigma_\chi^2}{k}\right),
\end{aligned}$$

where $\overline{\chi} = \frac{1}{k}\sum_{i=1}^{k}\chi_i$. We can now integrate out $m_\chi$, using the standard Gaussian integral

$$\int \mathcal{N}\left(\overline{\chi}|m_\chi, \frac{\sigma_\chi^2}{k}\right)\mathcal{N}(m_\chi|m^*, \sigma^{2*})dm_\chi \;\propto\; \mathcal{N}\left(\overline{\chi}|m^*, \sigma^{2*} + \frac{\sigma_\chi^2}{k}\right).$$

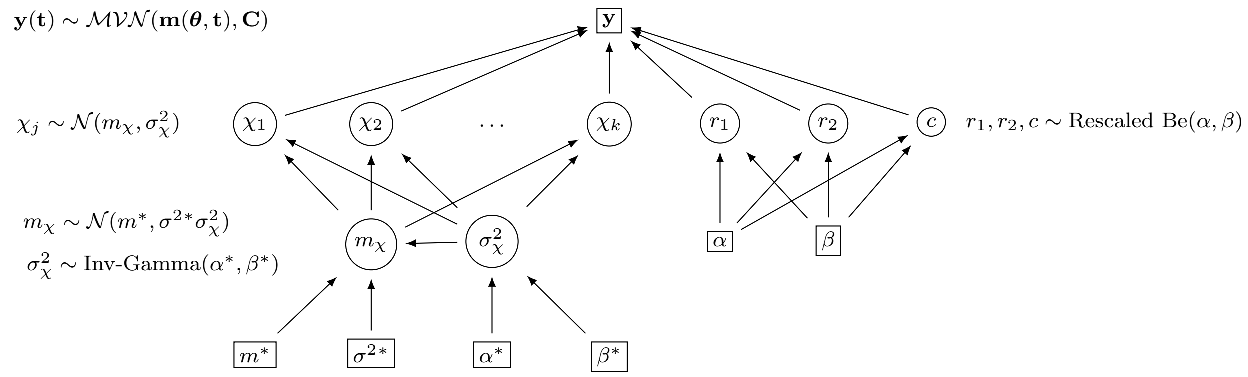


Figure 3: Modified Bayesian Hierarchical model to potentially enable computationally efficient inference, i.e. the prior is modified according to eq (19)), which corresponds to an additional edge from $\sigma^2_\chi$ to $m_\chi$ (to be compared to Figure 2 in the main paper). The model is used for vessel-specific stiffness analysis. The data, denoted by $\mathbf{y(t)}$, are assumed to follow a multivariate normal distribution with mean $\mathbf{m}(\boldsymbol{\theta}, \mathbf{t})$ and covariance matrix $\mathbf{C}$. If iid errors are assumed, $\mathbf{C}$ is a diagonal matrix, $\mathbf{C} = \sigma^2\mathbf{I}$ (where $\sigma^2$: error variance and $\mathbf{I}$: identity matrix), and if correlated errors are assumed, $\mathbf{C}$ is a full matrix. The biophysical parameters, $\boldsymbol{\theta} = (\chi_1, \dots \chi_k, r_1, r_2, c)$ (described in the main paper), and the hyperparameters, $m_\chi, \sigma^2_\chi$, are apriori drawn from the distributions indicated in the graphical model. The circle represents variable quantities, which are inferred using MCMC, and the rectangle stands for fixed quantities.

This gives:

$$
\begin{aligned}
p(\sigma^2_\chi|\text{everything else except } m_\chi) \quad &\propto \quad \left(\frac{1}{\sigma^2_\chi}\right)^{\alpha^*+1+(k-1)/2} \exp\left(\frac{-\beta^*}{\sigma^2_\chi}\right) \exp\left(\frac{-1}{2\sigma^2_\chi}\sum_{i=1}^{k}(\chi_i - \overline{\chi})^2\right) \\
&\quad \mathcal{N}\left(\overline{\chi}|m^*, \sigma^{2*} + \frac{\sigma^2_\chi}{k}\right) \\
&\propto \quad \left(\frac{1}{\sigma^2_\chi}\right)^{\alpha^*+1+(k-1)/2} \exp\left(\frac{-\beta^*}{\sigma^2_\chi}\right) \exp\left(\frac{-1}{2\sigma^2_\chi}\sum_{i=1}^{k}(\chi_i - \overline{\chi})^2\right) \\
&\quad \left(\frac{1}{\sigma^{2*} + \frac{\sigma^2_\chi}{k}}\right)^{1/2} \exp\left(\frac{-(\overline{\chi} - m^*)^2)}{2(\sigma^{2*} + \frac{\sigma^2_\chi}{k})}\right).
\end{aligned}
$$

Hence, we can integrate out $m_\chi$ analytically to get the marginal distribution in closed form. However, due to the additive term $\sigma^{2*}$ in the denominator of the last two factors, the distribution is *not* in the family of inverse-gamma distributions, and we therefore cannot sample $\sigma^2_\chi$ from it directly. In order to proceed, we would have to set up a slice sampling or Metropolis-Hastings scheme. This is will almost certainly lose the efficiency gained from collapsing.

*5.2.3. How to get the collapsed Gibbs sampler to work*

We follow studies in [19, 20], that use a trick to enable collapsing, i.e. we modify the prior as follows:

$$
\mathcal{N}(m_\chi|m^*, \sigma^{2*}) \;\rightarrow\; \mathcal{N}(m_\chi|m^*, \sigma^{2*}\sigma^2_\chi). \tag{19}
$$

Graphically, this corresponds to introducing an additional edge from $\sigma^2_\chi$ to $m_\chi$ in the hierarchical model, see Figure 3.

$$p(m_\chi, \sigma^2_\chi|\text{everything else}) \propto \left(\frac{1}{\sigma^2_\chi}\right)^{\alpha^*+1}\left(\frac{1}{\sigma^2_\chi}\right)^{(k-1)/2}\exp\left(\frac{-\beta^*}{\sigma^2_\chi}\right)\exp\left(\frac{-1}{2\sigma^2_\chi}\sum_{i=1}^k(\chi_i-\overline{\chi})^2\right)$$
$$\mathcal{N}(m_\chi|m^*, \sigma^{2*}\sigma^2_\chi)\mathcal{N}\left(\overline{\chi}|m_\chi, \frac{\sigma^2_\chi}{k}\right).$$

Integrating out $m_\chi$ now gives:

$$\int \mathcal{N}\left(\overline{\chi}|m_\chi, \frac{\sigma^2_\chi}{k}\right)\mathcal{N}(m_\chi|m^*, \sigma^{2*}\sigma^2_\chi)dm_\chi \propto \mathcal{N}\left(\overline{\chi}|m^*, \sigma^2_\chi\left[\sigma^{2*}+\frac{1}{k}\right]\right),$$

leading to

$$\begin{aligned}
& p(\sigma^2_\chi|\text{everything else except for } m_\chi) \\
\propto\; & \left(\frac{1}{\sigma^2_\chi}\right)^{\alpha^*+1}\left(\frac{1}{\sigma^2_\chi}\right)^{(k-1)/2}\exp\left(\frac{-\beta^*}{\sigma^2_\chi}\right)\exp\left(\frac{-1}{2\sigma^2_\chi}\sum_{i=1}^k(\chi_i-\overline{\chi})^2\right) \\
& \mathcal{N}\left(\overline{\chi}|m^*, \sigma^2_\chi\left[\sigma^{2*}+\frac{1}{k}\right]\right) \\
\propto\; & \left(\frac{1}{\sigma^2_\chi}\right)^{\alpha^*+1+(k-1)/2}\exp\left(\frac{-\beta^*}{\sigma^2_\chi}\right)\exp\left(\frac{-1}{2\sigma^2_\chi}\sum_{i=1}^k(\chi_i-\overline{\chi})^2\right) \\
& \left(\frac{1}{\sigma^2_\chi}\right)^{1/2}\exp\left(\frac{-1}{2\sigma^2_\chi}\left[\sigma^{2*}+\frac{1}{k}\right]^{-1}(\overline{\chi}-m^*)^2\right) \\
=\; & \left(\frac{1}{\sigma^2_\chi}\right)^{\alpha^*+1+k/2}\exp\left\{\frac{-1}{\sigma^2_\chi}\left(\beta^*+\frac{1}{2}\sum_{i=1}^k(\chi_i-\overline{\chi})^2\right.\right. \\
& \left.\left.+\frac{1}{2}\left[\sigma^{2*}+\frac{1}{k}\right]^{-1}(\overline{\chi}-m^*)^2\right)\right\}.
\end{aligned}$$

Hence,

$$p(\sigma^2_\chi|\text{everything else except for } m_\chi) \propto \mathcal{IG}\left(\sigma^2_\chi\middle|\alpha^*+\frac{k}{2}, \left[\beta^*+\frac{1}{2}\sum_{i=1}^k(\chi_i-\overline{\chi})^2+\frac{1}{2}\left\{\sigma^{2*}+\frac{1}{k}\right\}^{-1}(\overline{\chi}-m^*)^2\right]\right). \tag{20}$$

This could give a faster and more efficient sampling scheme than the original naive Gibbs sampler, following

- $p(m_\chi|m^*, \sigma^{2*}, \chi_1, \ldots, \chi_k, \sigma^2_\chi)$ in eq (17),
- $p(\sigma^2_\chi|\alpha^*, \beta^*, \chi_1, \ldots, \chi_k, m^*, \sigma^{2*})$ in eq (20),
- $p(\boldsymbol{\chi}, r_1, r_2, c|m_\chi, \sigma^2_\chi, \mathbf{y}, \alpha, \beta)$.

*5.2.4. Final improvement: eliminating the need for Gibbs sampling*

However, we can improve mixing and convergence of the sampler even further with another round of collapsing. To simplify the notation, let $\Psi[.]$ denote all the 'prior' random variables (i.e. downstream of the data $\mathbf{y}$) except those included in the bracket. So $p(\sigma^2_\chi|\text{everything else except for } m_\chi) = p(\sigma^2_\chi|\Psi[m_\chi, \sigma^2_\chi])$. Here, the additional condition 'downstream of the data $\mathbf{y}$' is redundant, because $\mathbf{y}$ is not included in the

Markov blanket of $\sigma_\chi^2$. However, this additional condition does make a difference when looking at the distribution of the vector of stiffness parameters, $\boldsymbol{\chi} = (\chi_1, \ldots, \chi_k)$. Now, take the previously derived expression for $p(\sigma_\chi^2|\Psi[m_\chi, \sigma_\chi^2])$ in eq (20), keep the terms that depend on $\sigma_\chi^2$ and $\boldsymbol{\chi} = (\chi_1, \ldots, \chi_k)$, ignore all the other terms (because they will be absorbed in the normalization constant), and we get:

$$p(\sigma_\chi^2, \boldsymbol{\chi}|\Psi[m_\chi, \sigma_\chi^2]) \propto \left(\frac{1}{\sigma_\chi^2}\right)^{\alpha^*+1+k/2} \exp\left\{\frac{-1}{\sigma_\chi^2}\left(\beta^* + \frac{1}{2}\sum_{i=1}^{k}(\chi_i - \overline{\chi})^2 + \frac{1}{2}\left[\sigma^{2*} + \frac{1}{k}\right]^{-1}(\overline{\chi} - m^*)^2\right)\right\}.$$

Now marginalise over $\sigma_\chi^2$,

$$p(\boldsymbol{\chi}|\Psi[m_\chi\, \sigma_\chi^2]) = \int_0^\infty p(\sigma_\chi^2, \boldsymbol{\chi}|\Psi[m_\chi, \sigma_\chi^2])d\sigma_\chi^2$$

and make use of the gamma integral

$$\int_0^\infty \left(\frac{1}{x}\right)^{a+1} \exp\left(\frac{-b}{x}\right) dx \;=\; \frac{\Gamma(a)}{b^a}.$$

This leads to

$$p(\boldsymbol{\chi}|\Psi[m_\chi, \sigma_\chi^2]) \propto \frac{1}{\left(\beta^* + \frac{1}{2}\sum_{i=1}^{k}(\chi_i - \overline{\chi})^2 + \frac{1}{2}\left[\sigma^{2*} + \frac{1}{k}\right]^{-1}(\overline{\chi} - m^*)^2\right)^{(\alpha^*+k/2)}}. \tag{21}$$

The upshot is that $\sigma_\chi^2$ and $m_\chi$ have effectively been eliminated altogether. So the corresponding sampling steps of both the naive and the collapsed Gibbs sampler are no longer needed. All we have to do is run a standard MCMC scheme for $\boldsymbol{\chi}$, with the likelihood given by $p(\mathbf{y}|\boldsymbol{\chi}, r_1, r_2, c)$, the prior of $\{r_1, r_2, c\}$ taken from our hierarchical Bayesian model, and the prior of $\boldsymbol{\chi}$ given by eq (21) (where the unknown normalization constant cancels out in the Metropolis-Hastings ratio).

## 6. Posterior inference with conventional Bayesian methods (MCMC)

This section compliments Section 4.4 in the main paper.

The posterior distribution is computed as

$$p(\boldsymbol{\theta}, \boldsymbol{\eta}|\mathbf{y}) \propto p(\mathbf{y}|\boldsymbol{\theta}, \boldsymbol{\eta})p(\boldsymbol{\theta}, \boldsymbol{\eta}), \tag{22}$$

where $\boldsymbol{\theta}$ are the biophysical parameters and $\boldsymbol{\eta}$ are the error parameters.

In this study, we pursue Bayesian inference based on sampling the biophysical parameters from their posterior distribution. The posterior in eq (22) is unavailable in closed form, hence we employ MCMC techniques, i.e. the Adaptive Metropolis (AM) algorithm [21].

In the correlated errors analysis, the GP neural network hyperparameters are jointly sampled with the biophysical parameters from their joint posterior distribution using the AM algorithm. We fix the noise variance of the residuals $\sigma_n^2$ to a very small value ($10^{-6}$), as supported by preliminary analysis findings, to avoid numerical instabilities from the matrix inversion $\mathbf{C}^{-1}$ in eq (16).

In the iid errors analysis, we sample the measurement noise variance in a Gibbs step [22] from the conditional posterior distribution, $p(\sigma^2|\boldsymbol{\theta}, \mathbf{y})$, which is available in closed form:

$$p(\sigma^2|\boldsymbol{\theta}, \mathbf{y}) = \mathcal{IG}\left(\frac{n}{2} + a, 0.5\sum_{i=1}^{n}(y_i - m_i(\boldsymbol{\theta}))^2 + b\right), \tag{23}$$

where $a = 0.001$ and $b = 0.001$.

The choice of the priors in the Bayesian hierarchical model used in the main paper leads to closed-form posterior distributions for the hyperparameters $m_\chi, \sigma_\chi^2$, see eqns (24) and (25), which can be sampled using

the Gibbs algorithm [22]. Thus, posterior inference in the hierarchical model, summarised in Algorithm 1, proceeds by iterative sampling from eqns (24), (25) and (26).

$$p(m_\chi|m^*, {\sigma^2}^*, \boldsymbol{\chi}, \sigma_\chi^2) = \mathcal{N}\left(\frac{\frac{m^*}{{\sigma^2}^*} + \frac{1}{\sigma_\chi^2}\sum_{i=1}^k \chi_i}{\frac{1}{{\sigma^2}^*} + \frac{k}{\sigma_\chi^2}}, \left[\frac{1}{{\sigma^2}^*} + \frac{k}{\sigma_\chi^2}\right]^{-1}\right), \tag{24}$$

$$p(\sigma_\chi^2|\alpha^*, \beta^*, \boldsymbol{\chi}, m_\chi) = \mathcal{IG}\left(\alpha^* + \frac{k}{2}, \beta^* + 0.5\sum_{i=1}^k(\chi_i - m_\chi)^2\right), \tag{25}$$

$$p(\boldsymbol{\chi}, \psi_1, \psi_2, c|m_\chi, \sigma_\chi^2, \mathbf{y}, \alpha, \beta), \tag{26}$$

where the sampling of $\boldsymbol{\chi}, \psi_1, \psi_2, c$ cannot be done analytically and follows a Metropolis-Hastings within Gibbs scheme. Also, $k = |\boldsymbol{\chi}|$ is the cardinality of the stiffness parameter vector.

**Algorithm 1** Adaptive Metropolis with Gibbs sampling for the hyperparameters of the hierarchical model

1: Define N: number of MCMC samples.
2: **for** i=1:N **do**
3: Given $m_\chi^{(i-1)}$ and ${\sigma^2}_\chi^{(i-1)}$, use the AM algorithm to draw $(\boldsymbol{\chi}^{(i)}, \psi_1^{(i)}, \psi_2^{(i)}, c^{(i)})$ from the approximate posterior distribution derived from eqns (26) and (22) with the likelihood in Section 4.1 in the main paper and priors in Section 4.3
4: Given $\boldsymbol{\chi}^{(i)}$ and ${\sigma^2}_\chi^{(i-1)}$, draw $m_\chi^{(i)}$ in a Gibbs step [22] using the conditional distributions in eq (24)
5: Given $\boldsymbol{\chi}^{(i)}$ and $m_\chi^{(i)}$, draw ${\sigma^2}_\chi^{(i)}$ in a Gibbs step [22] using the conditional distributions in eq (25)
6: **end for**

The Bayesian hierarchical framework can reveal if vessel-specific stiffness parameters are needed: if $\sigma_\chi^2 \to 0$, then all stiffness parameters take values close to $m_\chi$ with high probability, encouraging a common stiffness parameter, while $\sigma_\chi^2 \to \infty$ will be evidence that the stiffness parameters are statistically independent of each other, implying vessel-specific stiffness values.

## 7. Posterior inference with emulation Bayesian methods (MCMC)

This section highlights how we propose to perform parameter estimation and uncertainty quantification in expensive models, such as the fluid-dynamics model under consideration, by using a novel combination of state-of-the-art statistical inference techniques in a timely manner, bringing us closer to achieving real-time treatment planning. Firstly, we discuss how an MCMC approach can be coupled with emulation using GPs to achieve considerable computational speed-up. To do this, we follow the work by Rasmussen in [23], and make various modifications inspired from the statistical literature (e.g. Delayed Acceptance MCMC [24, 25]) which have the potential to further reduce the computational costs. In [23], Rasmussen employs the HMC algorithm, while we choose to utilise the AM algorithm since pilot studies have indicated that the N-steps ahead GP AM algorithm provides a better trade-off between efficiency and accuracy compared to the GP HMC algorithm for our fluid-dynamics model. In addition, there are regions in the parameter space which break the physical assumptions of the fluid-dynamics model, and to avoid entering the infeasible parameter domains, we extend Rasmussen's algorithm to incorporate a GP classifier which automatically excludes these regions. Below we give a brief method summary.

### *7.1. Emulator-based data likelihood*

We use GP regression to emulate the data log likelihood, $\mathcal{L}$, rather than the output from the PDEs, the blood pressure (i.e. we build a surrogate for the log likelihood). The input variables are the PDE model parameters (and the error model parameters if correlated errors are assumed, see below). Details for the GP model set-up can be found in Section 8.1.

We assume the data likelihood to be Gaussian.

- Independent and identically distributed (iid) errors:

$$\tilde{p}(\mathbf{y}|\boldsymbol{\theta}, \sigma^2) = \left(\frac{1}{\sqrt{2\pi\sigma^2}}\right)^n \exp\left(-\frac{\tilde{\mathcal{S}}(\boldsymbol{\theta})}{2\sigma^2}\right), \tag{27}$$

  where $\tilde{\mathcal{S}}(\boldsymbol{\theta})$ is the value of the residual sum-of-squares,

$$\tilde{\mathcal{S}}(\boldsymbol{\theta}) = \sum_{i=1}^{n}(y_i - m_i(\boldsymbol{\theta}))^2, \tag{28}$$

  predicted by the emulator (the GP posterior predictive mean – see eq. (7)) for the particular $(\boldsymbol{\theta}, \sigma^2)$ parameter vector. We note that $\mathbf{m}(\boldsymbol{\theta})$ is the vector of pressure predictions from the mathematical model, $\mathbf{y}$ is the vector of temporal pressure measurements, $n$ is the number of time points, and $\sigma^2$ is the noise variance.

- Correlated errors:

$$\tilde{p}(\mathbf{y}|\boldsymbol{\theta}, \mathbf{C}(\boldsymbol{\eta})) = \exp(\tilde{\mathsf{L}}(\boldsymbol{\theta}, \boldsymbol{\eta})), \tag{29}$$

  where $\tilde{\mathsf{L}}(\boldsymbol{\theta}, \boldsymbol{\eta})$ is the log likelihood value predicted by the emulator (the GP posterior predictive mean – eq. (7)) for the particular $(\boldsymbol{\theta}, \boldsymbol{\eta})$ parameter vector (here $\boldsymbol{\eta} = (w, b)$, where $w$ and $b$ are the neural network hyperparameters (see eq (5)). Additionally, $\mathbf{C}$ is the covariance matrix of the errors.

*7.2. Emulator-based prior distribution*

We use the GP classifier to avoid moving into areas of the parameter space where the physical assumptions of the fluid-dynamics model are violated. The GP classifier will provide the probability of a successful simulation (implementation details in Section 8.1.), and it is incorporated in the MCMC simulation through a modified prior distribution:

$$\tilde{p}(\boldsymbol{\theta}) \;=\; p(\boldsymbol{\theta})p(\lambda = 1|\boldsymbol{\theta}, \mathcal{H})/Z, \tag{30}$$

where $\lambda$ denotes a binary variable to indicate if the parameter vector $\boldsymbol{\theta}$ falls into a valid regime ($\lambda = 1$) or invalid regime ($\lambda = 0$), $\mathcal{H}$ is the list of training points for the classifier, and $Z = \int p(\boldsymbol{\theta})p(\lambda = 1|\boldsymbol{\theta}, \mathcal{H})d\boldsymbol{\theta}$ is a normalisation constant, which cancels out in the Metropolis-Hastings acceptance/rejection step. We note that the original prior distribution $p(\boldsymbol{\theta})$ is given in the main paper. Additionally, the prior for the error parameters is the original one presented in the main paper.

*7.3. Posterior inference with Bayesian analysis*

The posterior distribution is computed as

$$\tilde{p}(\boldsymbol{\theta}, \boldsymbol{\eta}|\mathbf{y}) \propto \tilde{p}(\mathbf{y}|\boldsymbol{\theta}, \boldsymbol{\eta})\tilde{p}(\boldsymbol{\theta})p(\boldsymbol{\eta}), \tag{31}$$

where $\boldsymbol{\theta}$ are the biophysical (PDE) parameters and $\boldsymbol{\eta}$ are the error parameters, i.e. $\eta = \sigma^2$ (noise variance) for iid errors, and $\boldsymbol{\eta} = (w, b)$ (GP neural network hyperparameters) for correlated errors.

We combine the MCMC algorithm with emulation and classification using GPs, and we run these in a Delayed Acceptance (DA) framework as part of the DA-GP-MCMC algorithm [23, 26]. The Delayed Acceptance MCMC algorithm, proposed in [24, 25], is based on a two-stage scheme: once a new point is proposed, in the first stage it is subject to a MH acceptance step based on the emulator (i.e. surrogate model in (eq 32)); if the point is accepted, it goes through a second stage acceptance step based on the simulator (i.e. PDE-based model in (eq 33)). The first stage can be run a number of times $N, N > 1$, i.e. we propose and accept/reject $N$ points in the surrogate space of the log posterior distribution. The $N^{\text{th}}$ point is subject to an accept/reject point based on the simulator. More details can be found in our recent study [26], where we show that this approach achieves computational savings over the classical 1-step version of it ($N = 1$). The N-steps ahead DA-GP-MCMC algorithm is summarised in Section 7.3.1. The emulation approach is best suited for low to moderate parameter dimensionality. As the parameter dimensionality increases, the training points provide a sparser coverage of the parameter space, which in turn affects the accuracy of the emulator.

*7.3.1. N-steps ahead DA-GP-MCMC algorithm*

The N-steps ahead DA-GP-MCMC algorithm proceeds as follows:

- *Initial design stage.* Starting from a space filling design in parameter space, e.g. Sobol sequence [27], integrate the PDEs numerically for each parameter vector to get the true log likelihood and the corresponding PDE success label. Use these points to build a GP emulator (surrogate model) for the log likelihood and a GP classifier indicating the valid parameter combinations. The GP models are built on a compact parameter space, where the lower and upper limits are decided in advance.

- *Exploratory phase.* Gather information about the target distribution by running MCMC on the surrogate log posterior of the PDE parameters; the proposed point is subject to a MH accept/reject step, for which the simulator is called, i.e. the PDEs are integrated numerically – see the pseudocode in Algorithm 2 for one iteration of the algorithm. The emulator and the classifier are sequentially refined (optimum covariance hyperparameters are found by maximisation of the log marginal likelihood of the hyperparameters) as new points are accepted. The points which yield sucessful PDE integration and have subsequently been accepted in the MCMC simulation are added as further training points for the emulator. All points, regardless of whether or not they provide successful PDE integration and/or are accepted in the MCMC are added as training points for the classifier. Implementation details can be found in Section 8.2. Following [23], we set the emulated approximate log posterior distribution of the MCMC algorithm to

$$\log \tilde{p}(\boldsymbol{\theta}, \boldsymbol{\eta}|\mathbf{y}) \propto \Big(\mathbb{E}(f(\boldsymbol{\theta}, \boldsymbol{\eta})|D) + \sqrt{\mathrm{var}(f(\boldsymbol{\theta}, \boldsymbol{\eta})|D)}\Big) + \log \tilde{p}(\boldsymbol{\theta}) + \log p(\boldsymbol{\eta}).$$

  Here $\tilde{p}(\boldsymbol{\theta})p(\boldsymbol{\eta})$ is the prior distribution, $f(.)$ is the emulated log likelihood function, $\mathbb{E}(f(\boldsymbol{\theta}, \boldsymbol{\eta})|D)$ is the GP posterior predictive mean given the training points $D$ (see eq. (7)) and $\sqrt{\mathrm{var}(f(\boldsymbol{\theta}, \boldsymbol{\eta})|D)}$ is the GP posterior predictive standard deviation (see eq. (8)) for the log likelihood of the physiological data at unseen parameter configurations $\boldsymbol{\theta}, \boldsymbol{\eta}$ conditional on the training points $D$. This drives the exploration into regions with high posterior probability (large value of $\mathbb{E}(.)$) or high uncertainty (large value of $\sqrt{\mathrm{var}(.)}$). If $\sqrt{\mathrm{var}(.)}>3$ along the trajectory, the simulation is stopped prematurely before reaching the end of the trajectory, as the algorithm steps into a region of high uncertainty, where the GP needs to be further trained. The log likelihood is computed at this point by numerically solving the PDEs of the biophysical model, and the corresponding success label is obtained. The exploratory phase is run until high accuracy of the emulator and the classifier is reached (as quantified by GP diagnostics [28]).

- *Sampling phase.* Use the emulator and the classifier created in the exploratory phase to draw samples from the target distribution using MCMC – see the pseudocode in Algorithm 2, where the PDE model parameters and the GP neural network hyperparameters (error model parameters) are jointly sampled. At this stage, the emulator and the classifier are no longer updated. We set the emulated approximate log posterior distribution of the MCMC algorithm to

$$\log \tilde{p}(\boldsymbol{\theta}, \boldsymbol{\eta}|\mathbf{y}) \propto \mathbb{E}(f(\boldsymbol{\theta}, \boldsymbol{\eta})|D) + \log \tilde{p}(\boldsymbol{\theta}) + \log p(\boldsymbol{\eta}).$$

  Note that the numerator in the first term is the expected log likelihood of the data, and the final two terms are the log prior. The end point of the trajectory is subject to a 2-stage DA Metropolis-Hastings accept/reject step, based on the simulator, see Algorithm 2. The rejection rate is monitored, and this indicates how well the GP emulator has captured the log posterior density. A large number of rejections calls for an extension of the exploratory phase.

Note that the proposal distribution $q(.|.)$ in Algorithm 2 is that of an Adaptive Metropolis algorithm [21], i.e. a multivariate normal distribution centred at the current point, with covariance matrix adapted based on the past posterior samples.

**Algorithm 2** One iteration of the N-steps ahead Delayed Acceptance GP-MCMC algorithm

1: Define a probabilistic model $p(\mathbf{y}|\boldsymbol{\theta}, \boldsymbol{\eta})$, where $\mathbf{y}$ are the data, $\boldsymbol{\theta}$ are the PDE model parameters with prior probability $p(\boldsymbol{\theta})$, and $\boldsymbol{\eta}$ are the error parameters with prior probability $p(\boldsymbol{\eta})$ . Let $\tilde{p}(\mathbf{y}|\boldsymbol{\theta})$ denote a computationally cheap surrogate model, and $\tilde{p}(\boldsymbol{\theta})$ is the modified prior (see eq (30)). The corresponding posterior probabilities are given by $p(\boldsymbol{\theta}, \boldsymbol{\eta}|\mathbf{y}) \propto p(\mathbf{y}|\boldsymbol{\theta}, \boldsymbol{\eta})p(\boldsymbol{\theta})p(\boldsymbol{\eta})$ and $\tilde{p}(\boldsymbol{\theta}, \boldsymbol{\eta}|\mathbf{y}) \propto \tilde{p}(\mathbf{y}|\boldsymbol{\theta}, \boldsymbol{\eta})\tilde{p}(\boldsymbol{\theta})p(\boldsymbol{\eta})$.

2: Define N: number of proposed points before the simulator is called.

3: **for** i=2:N **do**

4: Given the current parameter vector $(\boldsymbol{\theta}_{i-1}, \boldsymbol{\eta}_{i-1})$, draw new parameters $(\boldsymbol{\theta}_i, \boldsymbol{\eta}_i)$ from the proposal distribution $q(\boldsymbol{\theta}_i, \boldsymbol{\eta}_i|\boldsymbol{\theta}_{i-1}, \boldsymbol{\eta}_{i-1})$, and accept the move with the acceptance probability:

$$\alpha_1(\boldsymbol{\theta}_i, \boldsymbol{\eta}_i|\boldsymbol{\theta}_{i-1}, \boldsymbol{\eta}_{i-1}) = \min\left(1, \frac{\tilde{p}(\boldsymbol{\theta}_i, \boldsymbol{\eta}_i|\mathbf{y})q(\boldsymbol{\theta}_{i-1}, \boldsymbol{\eta}_{i-1}|\boldsymbol{\theta}_i, \boldsymbol{\eta}_i)}{\tilde{p}(\boldsymbol{\theta}_{i-1}, \boldsymbol{\eta}_{i-1}|\mathbf{y})q(\boldsymbol{\theta}_i, \boldsymbol{\eta}_i|\boldsymbol{\theta}_{i-1}, \boldsymbol{\eta}_{i-1})}\right). \tag{32}$$

5: **end for**

6: The final proposed parameter vector $(\boldsymbol{\theta}_N, \boldsymbol{\eta}_N)$ is subject to a second stage acceptance probability:

$$\alpha_2(\boldsymbol{\theta}_N, \boldsymbol{\eta}_N|\boldsymbol{\theta}_1, \boldsymbol{\eta}_1) = \min\left(1, \frac{p(\boldsymbol{\theta}_N, \boldsymbol{\eta}_N|\mathbf{y})}{p(\boldsymbol{\theta}_1, \boldsymbol{\eta}_1|\mathbf{y})} \frac{\tilde{p}(\boldsymbol{\theta}_1, \boldsymbol{\eta}_1|\mathbf{y})}{\tilde{p}(\boldsymbol{\theta}_N, \boldsymbol{\eta}_N|\mathbf{y})}\right). \tag{33}$$

## 8. Method implementation details

In this section we provide a few method implementation details.

### *8.1. GPs set-up*

For the GP regression model of the log likelihood, we used a squared exponential covariance function, and for the GP classifier we used a Matérn 3/2 covariance function, as chosen based on the data, via 1-fold cross-validation. More specifically, for the regression model, the cross-validation procedure used a score based on the absolute value of the deviation of the data $\mathbf{y}$ from the GP predictions $\hat{\mathbf{y}}$, i.e. $|\mathbf{y} - \hat{\mathbf{y}}|$. One log likelihood data point at a time was left out from the training set of the emulator, the log marginal likelihood was optimised with respect to the hyperparameters, the GP model thus obtained was used to predict the hold-out log likelihood point, and the prediction was compared to the hold-out point. The kernel which recorded the smallest deviation was the best. The procedure is similar for the GP classifier, the score used being based on the number of times that the classifier correctly classified the log likelihood points (e.g. if the probability of success for the point, as predicted by the classifier, is greater than 0.5 and the observation label is 1 (success), or the probability is below 0.5 and the observation label is 0 (failure), then the point has been correctly classified). In addition, we mention that for the classifier, the intractable integral in equation (12), which is required for obtaining the class probabilities, was approximated with expectation propagation; see Section 3.6 in [10] for details.

### *8.2. Parameter transformations*

To improve numerical stability and reduce round-off errors [29], the original parameters $\theta_i$, which are on different scales, were scaled in the order of 1 for building the GP emulator and GP classifier. In addition, the MCMC algorithm was run in this transformed parameter space, meaning that the parameter proposal was done in the transformed domain, see our previous study [29] for more details. The transformed parameters were mapped back via the inverse transformation into the original domain for the PDE simulator, i.e. when they were inserted into the mathematical PDE model.

### *8.3. Bayesian Hierarchical model for the stiffness – prior distribution*

We mentioned in the main paper (Section 4.3.1) that in the Bayesian Hierarchical model the hyper-hyperparameters $(m_\chi, \sigma_\chi^2, \alpha^*, \beta^*)$ take fixed values. Here we give details of how these fixed values were

chosen. The stiffness mean, $m_\chi$, was chosen to be within the physiological range via the fixed hyperparameters $m^*$ and $\sigma^{2*}$. The stiffness variance, $\sigma^2_\chi$, as chosen via the fixed hyperparameters $\alpha^*, \beta^*$, controls the spread around $m_\chi$, thus a 90% probability of the stiffness being inside the physiological range is controlled by $\sigma^2_\chi$.

For the iid errors analysis, we set $m^* = 51690$ (which is the posterior mean value obtained from the MCMC simulation for the constant stiffness model under the iid error assumption, see Table 4 in the main paper), $\sigma^{2*} = 1e + 08$, $\alpha^* = 3$, and $\beta^* = 1e + 09$.

For the correlated errors analysis, we set $m^* = 43075$ (which is the posterior mean value obtained from the MCMC simulation for the constant stiffness model under the correlation errors assumption, see Table 4 in the main paper), $\sigma^{2*} = 2.5e + 08$, $\alpha^* = 3$, and $\beta^* = 0.6e + 09$.

### *8.4. Error correlation parameters – prior ranges*

As mentioned in the main paper (Section 4.3.2), we chose a log uniform prior distribution for the GP neural network hyperparameters (error parameters used in the error correlation analysis), with the range chosen based on maximising the profile log likelihood. Here we give more details of how the range was chosen. The log uniform distribution was chosen to ensure a sufficiently large prior coverage. We fitted a GP to the residuals obtained by a difference between the measured data and the data generated from the mathematical model with the MAP estimate from the iid errors analysis. We then obtained the profile log likelihood for each hyperparameter, i.e. by varying one hyperparameter at once (within a wide range), we maximised the marginal log likelihood in eq (34) with respect to the other kernel hyperparameter. The maximised marginal log likelihood in eq (34) (called profile log likelihood) was plotted against the varying hyperparameter. This plot informed of the range that the varying hyperparameter should take. The range should cover a concave-looking profile log likelihood curve – the maximum of this curve is the maximum profile log likehood value [30], which should be included in the range; we exclude hyperparameter values from the range which give a small profile log likelihood value relative to the maximum value, e.g. below 10% of the maximum value.

$$\log p(\boldsymbol{\Gamma}|\mathbf{t}, \boldsymbol{\eta}, \sigma_n^2) = -\frac{n}{2}\log(2\pi) - \frac{1}{2}\log|\mathbf{K} + \sigma_n^2\mathbf{I}| - \frac{1}{2}\boldsymbol{\Gamma}^T(\mathbf{K} + \sigma_n^2\mathbf{I})^{-1}\boldsymbol{\Gamma}. \quad (34)$$

## 9. Additional results to those in the main paper

While in the main paper we show results for a few blood vessels, here we attach the complete results, for all 21 blood vessels.

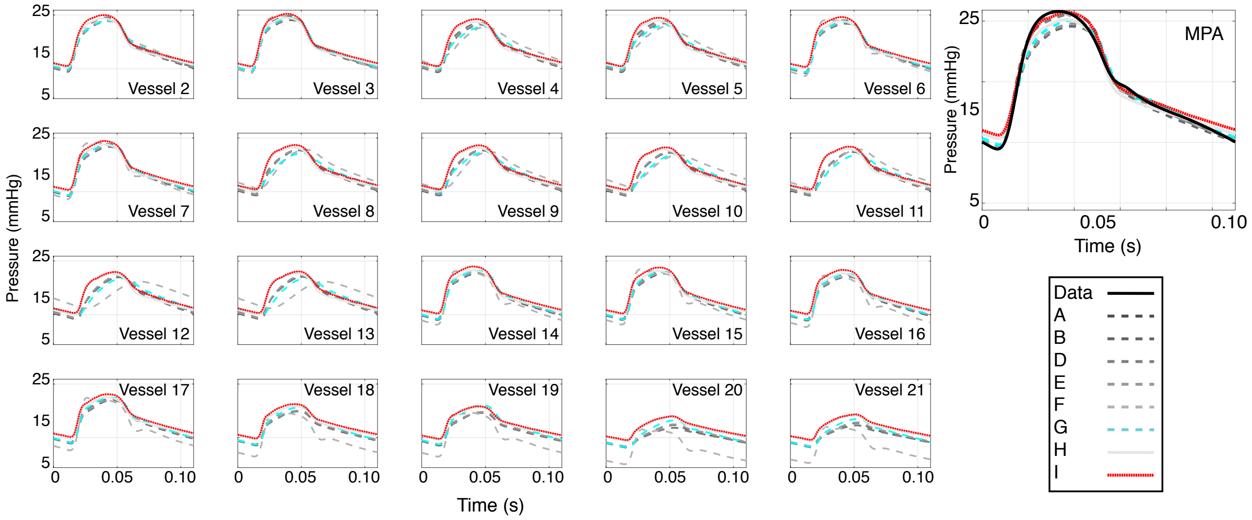


Figure 4: Pressure predictions obtained using the MCMC posterior samples for the parameters from all the models described in the main paper (Tables 1 and 2), which are denoted by A-I in the figure legend. We show the median pressure signal for 21 blood vessels in time. We superimpose the measured pressure data in the MPA. This figure corresponds to Figure 8 in the main paper.

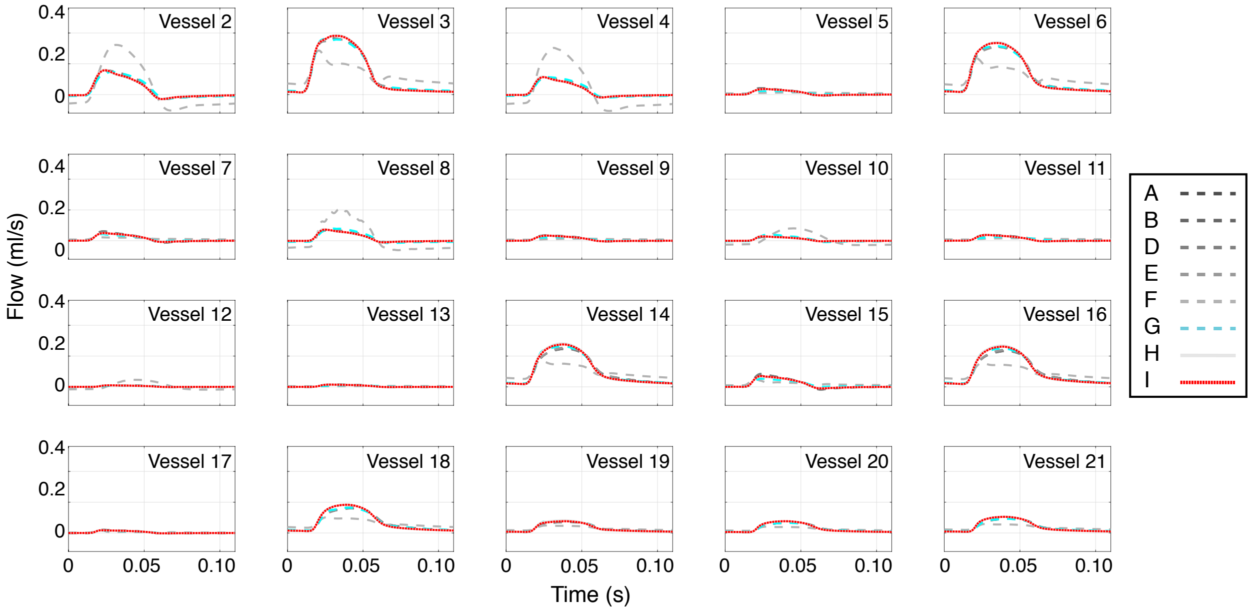


Figure 5: Flow predictions obtained using the MCMC posterior samples for the parameters from all the models described in the main paper (Tables 1 and 2), which are denoted by A-I in the figure legend. We show the median flow signal for blood vessels 2-21 in time. The MPA flow is used as inflow boundary condition for the PDEs. This figure corresponds to the left side subplots in Figure 9 of the main paper.

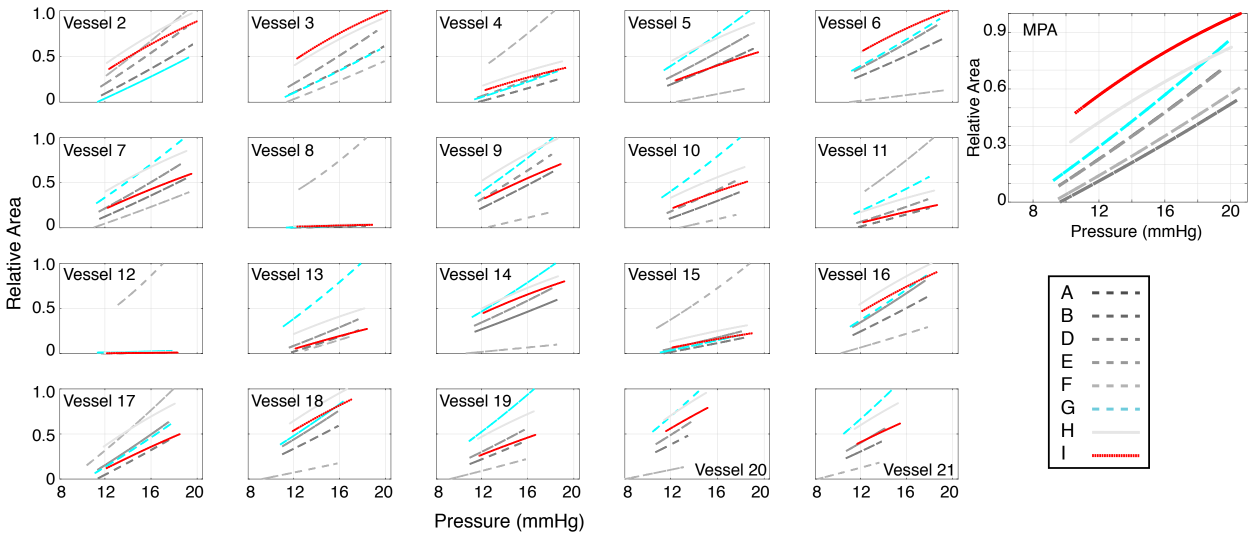


Figure 6: Pressure-Area predictions obtained using the MCMC posterior sample for the parameters from all the models described in the main paper (Tables 1 and 2), which are denoted by A-I in the figure legend. We show the median pressure prediction versus standardised cross-sectional area predictions for all 21 blood vessels. The area, $A_i$ is standardised per vessel $i$ to lie between [0,1] using the expression: $\frac{A_i - l_i}{u_i - l_i}$, where $l_i, u_i$ are the maximum and minimum area value for vessel $i$. $\mathbf{l} =$ [0.010, 0.003, 0.006, 0.003, 0.001, 0.004, 0.001, 0.002, 0.001, 0.002, 0.001, 0.001, 0.001, 0.002, 0.002, 0.002, 0.001, 0.002, 0.001, 0.002, 0.001]; $\mathbf{u} =$ [0.017, 0.005, 0.010, 0.006, 0.001, 0.007, 0.002, 0.032, 0.002, 0.003, 0.003, 0.035, 0.002, 0.005, 0.004, 0.004, 0.002, 0.004, 0.002, 0.003, 0.002]. This figure corresponds to the right side subplots in Figure 9 of the main paper.

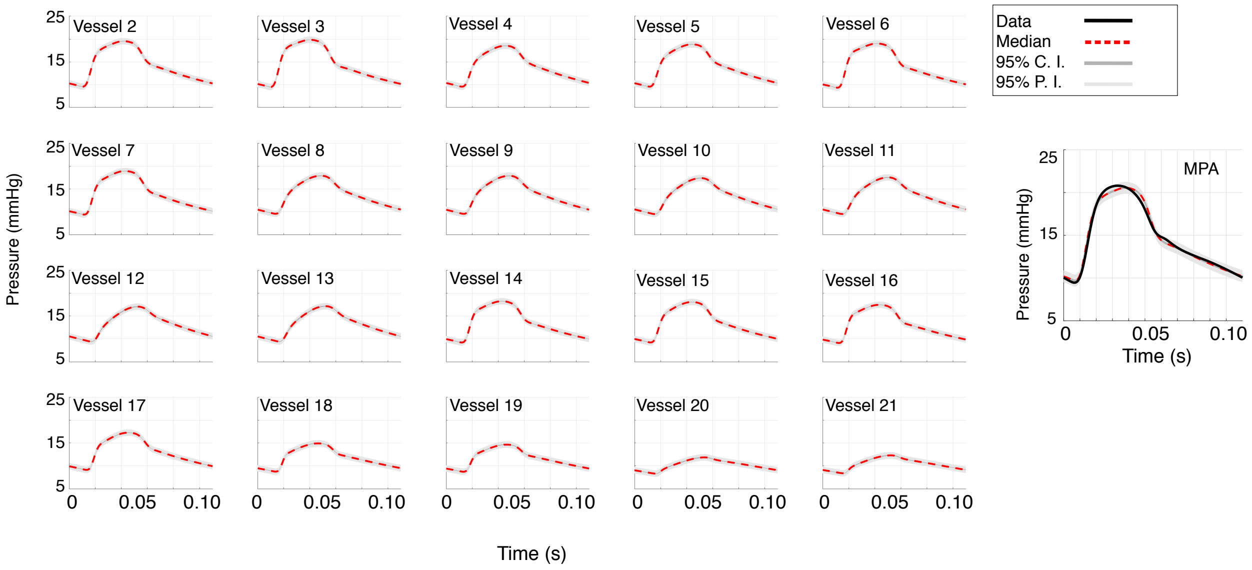


Figure 7: 95% credible intervals (C.I.) and prediction intervals (P.I.) for the pressure prediction in 21 vessels from the linear model with constant stiffness and no model mismatch (model A in Tables 1 and 2 of the main paper) obtained from MCMC posterior samples. We superimpose the measured pressure data in the MPA and the median prediction. This figure corresponds to the left column subplots in Figure 5 of the main paper.

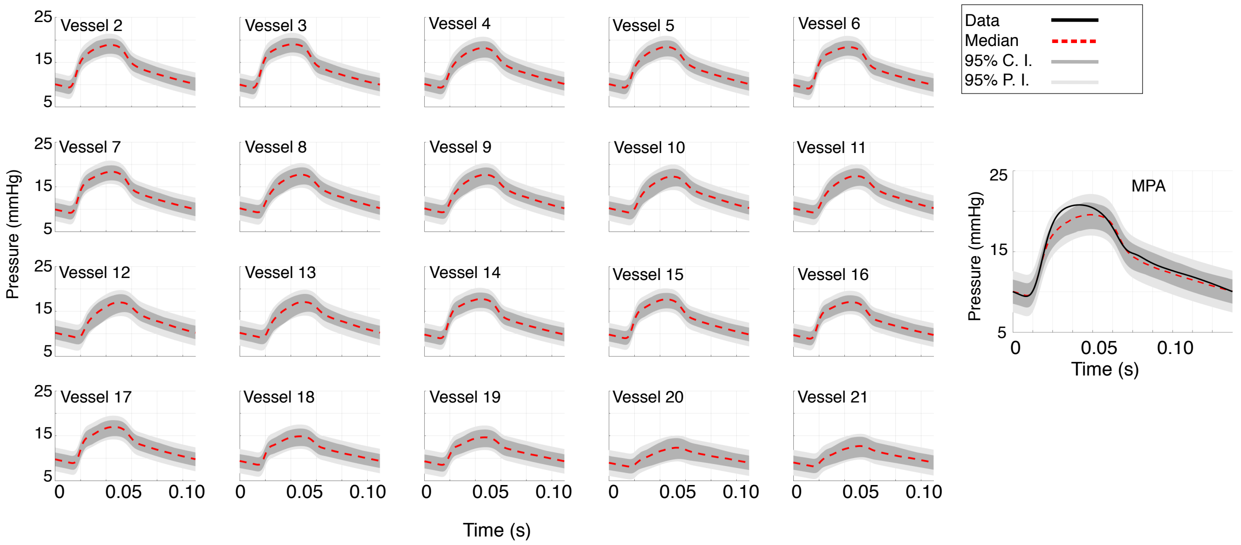


Figure 8: 95% credible intervals (C.I.) and prediction intervals (P.I.) for the pressure prediction in 21 vessels from the linear model with constant stiffness and model mismatch (model B in Tables 1 and 2 of the main paper) obtained from MCMC posterior samples. We superimpose the measured pressure data in the MPA and the median prediction. This figure corresponds to the centre column subplots in Figure 5 of the main paper.

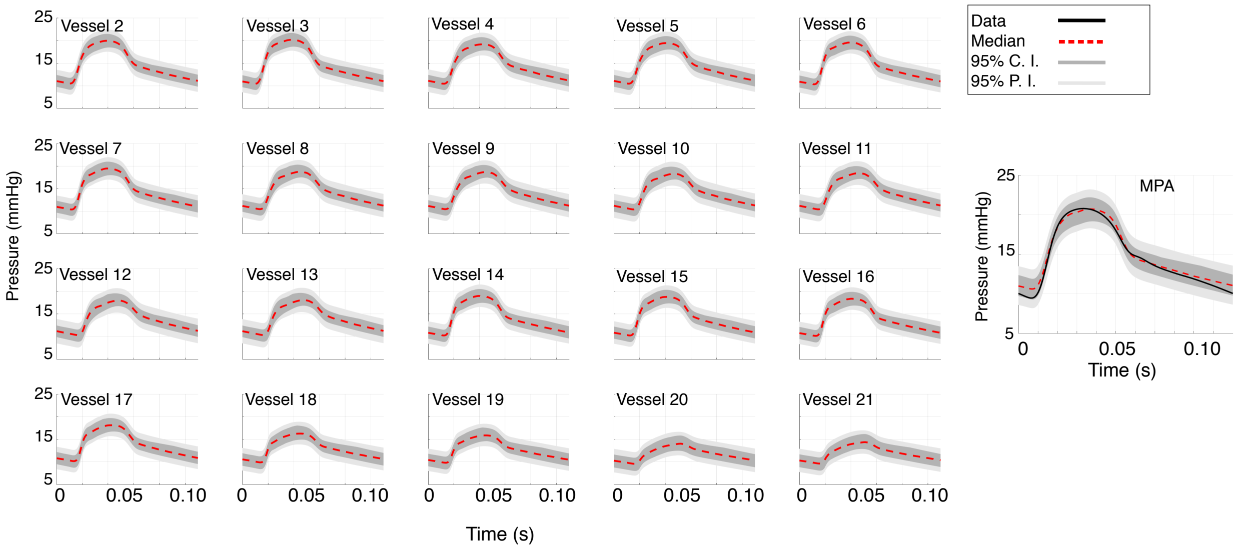


Figure 9: 95% credible intervals (C.I.) and prediction intervals (P.I.) for the pressure prediction in 21 vessels from the non-linear model with radius-dependent stiffness and model mismatch (model I in Tables 1 and 2 of the main paper) obtained from MCMC posterior samples. We superimpose the measured pressure data in the MPA and the median prediction. This figure corresponds to the right column subplots in Figure 5 of the main paper.

| Vessel | Radius | Model A | Model B | Model C | Model D | Model E | Model F | Model G | Model H | Model I |
|---|---|---|---|---|---|---|---|---|---|---|
| 1 | 0.05 | 14.7 (14.6 14.7)<br>(14.0 15.4) | 14.2 (12.6 15.7)<br>(11.7 16.7) | 14.0 (12.7 15.4)<br>(11.7 16.4) | 14.7 (14.6 14.8)<br>(14.0 15.4) | 14.4 (12.7 16.0)<br>(11.9 16.8) | 14.7 (14.6 14.7)<br>(14.3 15.1) | 14.6 (13.0 15.9)<br>(12.1 17.0) | 14.5 (13.2 16.1)<br>(12.1 16.9) | 15.0 (13.7 16.4)<br>(12.7 17.4) |
| 2 | 0.03 | 14.3 (14.2 14.3)<br>(13.5 15.0) | 13.8 (12.2 15.4)<br>(11.3 16.3) | 13.7 (12.3 15.0)<br>(11.3 16.1) | 14.3 (14.2 14.3)<br>(13.6 15.0) | 14.0 (12.3 15.6)<br>(11.5 16.5) | 14.4 (14.3 14.5)<br>(14.0 14.8) | 14.2 (12.5 15.6)<br>(11.7 16.6) | 14.2 (12.8 15.9)<br>(11.8 16.6) | 14.7 (13.4 16.1)<br>(12.3 17.1) |
| 3 | 0.04 | 14.3 (14.2 14.3)<br>(13.6 15.0) | 13.9 (12.3 15.4)<br>(11.3 16.3) | 13.7 (12.3 15.0)<br>(11.3 16.1) | 14.3 (14.2 14.3)<br>(13.6 15.0) | 14.0 (12.4 15.7)<br>(11.5 16.5) | 14.2 (14.2 14.3)<br>(13.8 14.7) | 14.2 (12.6 15.5)<br>(11.7 16.7) | 14.2 (12.9 15.9)<br>(11.8 16.6) | 14.7 (13.4 16.1)<br>(12.4 17.1) |
| 4 | 0.02 | 13.9 (13.8 13.9)<br>(13.1 14.6) | 13.5 (11.9 15.1)<br>(11.0 16.0) | 13.3 (11.9 14.7)<br>(11.0 15.8) | 13.9 (13.8 13.9)<br>(13.2 14.6) | 13.7 (12.0 15.3)<br>(11.1 16.2) | 14.2 (14.1 14.3)<br>(13.7 14.6) | 13.8 (11.9 15.4)<br>(11.1 16.3) | 14.0 (12.6 15.6)<br>(11.6 16.4) | 14.4 (13.1 15.8)<br>(12.0 16.8) |
| 5 | 0.01 | 14.0 (13.9 14.0)<br>(13.3 14.7) | 13.6 (12.0 15.2)<br>(11.1 16.1) | 13.4 (12.0 14.8)<br>(11.1 15.9) | 14.0 (13.9 14.0)<br>(13.3 14.7) | 13.8 (12.1 15.4)<br>(11.2 16.3) | 14.2 (14.1 14.3)<br>(13.8 14.6) | 13.9 (12.1 15.4)<br>(11.3 16.4) | 14.0 (12.7 15.7)<br>(11.6 16.5) | 14.5 (13.2 15.9)<br>(12.1 16.9) |
| 6 | 0.03 | 13.9 (13.8 13.9)<br>(13.1 14.6) | 13.5 (11.9 15.0)<br>(11.0 16.0) | 13.3 (11.9 14.7)<br>(11.0 15.7) | 13.8 (13.8 13.9)<br>(13.1 14.5) | 13.7 (12.0 15.3)<br>(11.1 16.1) | 13.6 (13.5 13.7)<br>(13.2 14.0) | 13.8 (12.1 15.1)<br>(11.3 16.3) | 13.9 (12.5 15.6)<br>(11.5 16.3) | 14.5 (13.1 15.8)<br>(12.1 16.9) |
| 7 | 0.02 | 13.8 (13.8 13.9)<br>(13.1 14.5) | 13.5 (11.9 15.0)<br>(10.9 16.0) | 13.3 (11.9 14.7)<br>(10.9 15.7) | 13.8 (13.8 13.9)<br>(13.1 14.5) | 13.7 (12.0 15.3)<br>(11.1 16.1) | 13.6 (13.5 13.7)<br>(13.2 14.1) | 13.8 (12.1 15.1)<br>(11.3 16.3) | 13.9 (12.5 15.6)<br>(11.5 16.3) | 14.4 (13.1 15.8)<br>(12.0 16.8) |
| 8 | 0.02 | 13.6 (13.5 13.6)<br>(12.9 14.3) | 13.3 (11.6 14.9)<br>(10.7 15.8) | 13.1 (11.6 14.5)<br>(10.7 15.5) | 13.6 (13.5 13.6)<br>(12.9 14.3) | 13.4 (11.7 15.1)<br>(10.9 15.9) | 14.1 (14.0 14.2)<br>(13.7 14.5) | 13.6 (11.4 15.2)<br>(10.8 16.2) | 13.8 (12.4 15.5)<br>(11.4 16.2) | 14.2 (12.8 15.6)<br>(11.8 16.6) |
| 9 | 0.02 | 13.5 (13.5 13.6)<br>(12.8 14.3) | 13.3 (11.5 14.9)<br>(10.7 15.8) | 13.1 (11.6 14.5)<br>(10.7 15.5) | 13.5 (13.5 13.6)<br>(12.8 14.2) | 13.4 (11.7 15.1)<br>(10.8 15.9) | 13.8 (13.7 14.0)<br>(13.4 14.3) | 13.6 (11.6 15.2)<br>(10.8 16.2) | 13.8 (12.3 15.5)<br>(11.3 16.2) | 14.2 (12.8 15.6)<br>(11.8 16.6) |
| 10 | 0.02 | 13.3 (13.2 13.4)<br>(12.6 14.0) | 13.0 (11.3 14.7)<br>(10.4 15.6) | 12.9 (11.4 14.3)<br>(10.4 15.3) | 13.3 (13.2 13.4)<br>(12.6 14.0) | 13.2 (11.5 14.9)<br>(10.6 15.7) | 14.0 (13.8 14.2)<br>(13.6 14.5) | 13.4 (10.9 15.1)<br>(10.4 16.1) | 13.6 (12.2 15.3)<br>(11.2 16.1) | 14.0 (12.6 15.5)<br>(11.6 16.4) |
| 11 | 0.02 | 13.4 (13.3 13.4)<br>(12.7 14.1) | 13.1 (11.4 14.7)<br>(10.5 15.6) | 12.9 (11.4 14.4)<br>(10.5 15.4) | 13.4 (13.3 13.5)<br>(12.7 14.1) | 13.3 (11.5 15.0)<br>(10.7 15.8) | 14.1 (14.0 14.2)<br>(13.7 14.5) | 13.4 (10.9 15.2)<br>(10.5 16.1) | 13.7 (12.2 15.4)<br>(11.2 16.1) | 14.1 (12.7 15.5)<br>(11.7 16.5) |
| 12 | 0.02 | 13.1 (13.0 13.2)<br>(12.4 13.8) | 12.9 (11.1 14.6)<br>(10.3 15.5) | 12.7 (11.2 14.2)<br>(10.3 15.2) | 13.1 (13.0 13.2)<br>(12.4 13.8) | 13.1 (11.3 14.8)<br>(10.5 15.6) | 13.8 (13.5 14.2)<br>(13.3 14.4) | 13.3 (10.7 15.0)<br>(10.3 16.0) | 13.5 (12.0 15.2)<br>(11.1 16.0) | 13.9 (12.5 15.3)<br>(11.5 16.3) |
| 13 | 0.01 | 13.2 (13.1 13.3)<br>(12.5 13.9) | 12.9 (11.2 14.6)<br>(10.3 15.5) | 12.8 (11.2 14.2)<br>(10.3 15.2) | 13.2 (13.1 13.3)<br>(12.5 13.9) | 13.1 (11.3 14.8)<br>(10.5 15.7) | 13.8 (13.5 14.2)<br>(13.3 14.4) | 13.3 (10.7 15.1)<br>(10.3 16.1) | 13.5 (12.1 15.3)<br>(11.1 16.0) | 13.9 (12.6 15.4)<br>(11.5 16.4) |
| 14 | 0.03 | 13.4 (13.4 13.5)<br>(12.7 14.1) | 13.1 (11.5 14.7)<br>(10.6 15.6) | 13.0 (11.6 14.3)<br>(10.6 15.4) | 13.4 (13.4 13.5)<br>(12.7 14.1) | 13.3 (11.6 15.0)<br>(10.8 15.8) | 12.8 (12.6 13.0)<br>(12.3 13.3) | 13.5 (11.8 14.9)<br>(11.0 15.9) | 13.7 (12.3 15.3)<br>(11.2 16.1) | 14.2 (12.8 15.6)<br>(11.8 16.6) |
| 15 | 0.02 | 13.4 (13.3 13.4)<br>(12.7 14.1) | 13.1 (11.4 14.6)<br>(10.5 15.6) | 12.9 (11.5 14.3)<br>(10.5 15.3) | 13.4 (13.3 13.4)<br>(12.7 14.1) | 13.3 (11.6 14.9)<br>(10.7 15.7) | 13.0 (12.8 13.1)<br>(12.5 13.4) | 13.4 (11.7 14.8)<br>(10.9 15.9) | 13.6 (12.2 15.3)<br>(11.2 16.0) | 14.1 (12.7 15.5)<br>(11.7 16.5) |
| 16 | 0.03 | 13.1 (13.0 13.1)<br>(12.3 13.8) | 12.8 (11.1 14.4)<br>(10.2 15.3) | 12.6 (11.2 14.0)<br>(10.2 15.1) | 13.0 (13.0 13.1)<br>(12.3 13.7) | 13.0 (11.2 14.6)<br>(10.4 15.4) | 12.2 (12.0 12.4)<br>(11.7 12.6) | 13.2 (11.3 14.6)<br>(10.6 15.7) | 13.4 (12.0 15.1)<br>(11.0 15.8) | 13.9 (12.5 15.3)<br>(11.5 16.3) |
| 17 | 0.02 | 13.0 (12.9 13.1)<br>(12.3 13.7) | 12.8 (11.1 14.3)<br>(10.2 15.3) | 12.6 (11.2 14.0)<br>(10.2 15.0) | 13.0 (12.9 13.1)<br>(12.3 13.7) | 12.9 (11.2 14.6)<br>(10.4 15.4) | 12.2 (11.8 12.4)<br>(11.6 12.7) | 13.1 (11.3 14.6)<br>(10.5 15.6) | 13.4 (11.9 15.1)<br>(10.9 15.8) | 13.8 (12.4 15.2)<br>(11.4 16.2) |
| 18 | 0.02 | 11.8 (11.7 11.9)<br>(11.1 12.5) | 11.7 (9.9 13.3)<br>(9.1 14.2) | 11.5 (10.1 13.0)<br>(9.1 14.0) | 11.8 (11.7 11.9)<br>(11.1 12.5) | 11.9 (10.0 13.6)<br>(9.3 14.4) | 10.3 (10.0 10.7)<br>(9.7 10.9) | 12.1 (10.0 13.9)<br>(9.3 14.8) | 12.5 (11.0 14.3)<br>(10.1 15.0) | 12.9 (11.5 14.4)<br>(10.5 15.4) |
| 19 | 0.02 | 11.6 (11.6 11.7)<br>(10.9 12.4) | 11.6 (9.8 13.2)<br>(9.0 14.1) | 11.4 (10.0 12.9)<br>(9.0 13.9) | 11.6 (11.6 11.7)<br>(10.9 12.3) | 11.8 (9.9 13.5)<br>(9.1 14.3) | 10.4 (9.9 10.7)<br>(9.7 11.0) | 12.2 (10.0 14.0)<br>(9.4 14.9) | 12.4 (10.9 14.2)<br>(10.0 14.9) | 12.7 (11.3 14.2)<br>(10.3 15.1) |
| 20 | 0.02 | 10.2 (10.1 10.3)<br>(9.5 10.9) | 10.3 (8.4 12.1)<br>(7.6 12.9) | 10.1 (8.6 11.7)<br>(7.7 12.7) | 10.2 (10.1 10.3)<br>(9.5 10.9) | 10.5 (8.5 12.3)<br>(7.8 13.1) | 8.1 (7.5 8.8)<br>(7.2 8.9) | 10.7 (8.3 13.2)<br>(7.5 14.0) | 11.5 (9.8 13.4)<br>(9.0 14.0) | 11.8 (10.3 13.3)<br>(9.4 14.3) |
| 21 | 0.02 | 10.4 (10.4 10.5)<br>(9.7 11.1) | 10.5 (8.6 12.3)<br>(7.8 13.1) | 10.3 (8.8 11.9)<br>(7.9 12.9) | 10.4 (10.4 10.5)<br>(9.7 11.1) | 10.7 (8.8 12.5)<br>(8.0 13.3) | 8.4 (7.8 9.1)<br>(7.6 9.2) | 10.9 (8.5 13.3)<br>(7.7 14.1) | 11.7 (10.0 13.5)<br>(9.2 14.2) | 12.0 (10.5 13.5)<br>(9.5 14.5) |

Table 1: Summary of the MCMC simulation results on measured data for each of the models considered, described in Tables 1 and 2 of the main paper. For each of the 21 blood vessels we indicate the average value over time of the median pressure waveform, as well as the average value over time of the $2.5^{\text{th}}$ and $97.5^{\text{th}}$ noise-free pressure waveform, which is the average 95% explanatory credible interval (CI) for the pressure data, and the $2.5^{\text{th}}$ and $97.5^{\text{th}}$ noisy pressure waveform, which is the average 95% predictive CI for the pressure data. While the explanatory CI is calculated based on the PDE model predictions, the predictive CI includes the error.

*Vessel-specific stiffness values.* Here we provide the vessel-specific stiffness values (median value and 95% credible interval), as obtained from running the Bayesian Hierarchical model on the physiological data. These values complement Table 4 in the main paper (rows 3 and 4 from bottom to top, column 5 from left to right, marked by '*').

For iid errors, $f_3$ : ($\times 10^4$) 4.93 (4.71, 5.10), 3.84 (3.61, 4.15), 6.10 (5.53, 7.10), 2.34 (2.10, 2.64), 10.4 (2.51, 18.1), 16.0 (9.16, 23.2), 6.27 (3.45, 10.9), 0.53 (0.51, 0.55), 9.95 (1.95, 17.00), 7.98 (6.36, 11.4), 2.29 (0.59, 7.00), 0.27 (0.23, 0.30), 5.54 (1.19, 14.36), 17.8 (9.77, 24.0), 1.94 (1.46, 2.85), 8.16 (4.67, 13.4), 3.47 (2.17, 11.3), 11.4 (6.30, 22.4), 7.63 (4.08, 12.5), 11.6 (5.51, 20.0), 8.44 (2.61,17.3).

For correlated errors, $f_3$ : ($\times 10^4$) 3.96 (3.57, 4.24), 5.76 (3.55, 9.32), 5.22 (4.27, 6.77), 4.46 (1.55, 12.9), 3.51 (0.52, 9.11), 4.13 (2.77, 6.01), 3.56 (1.86, 8.49), 4.47 (1.22, 12.24), 3.79 (0.73, 9.93), 2.94 (0.12, 11.9), 3.17 (0.26, 11.3), 2.62 (0.35, 11.2), 2.52 (0.23, 7.40), 3.43 (1.79, 8.15), 4.70 (2.28, 11.3), 4.15 (2.04, 11.6), 4.45 (2.21, 10.7), 4.05 (2.10, 12.9), 3.03 (1.23, 10.4), 3.30 (0.58, 10.6), 3.02 (0.41, 8.66).

We also provide the posterior median and 95% credible interval for the hyperpriors, $m_\chi$ and $\sigma^2_\chi$.

For iid errors, $m_\chi$ : ($\times 10^3$) 4.29 (-0.31, 10.9), and $\sigma^2_\chi$ : ($\times 10^9$) 5.81 (2.80, 13.9). For correlated errors, $m_\chi$ : ($\times 10^3$) 0.79 (-1.40, 3.22), and $\sigma^2_\chi$ : ($\times 10^9$) 1.8 (0.88, 4.10).

*Emulator efficiency.* Table 2 compares the efficiency of the standard MCMC sampler to that of MCMC with emulation using GPs, as described in Section 7, for the linear wall model with constant vessel stiffness (models B and C in Tables 1 and 2 of the main paper). Efficiency is quantified using the effective sample size (ESS) [31], and we observe that while the emulation method requires much fewer PDE evaluations (compare 5000 to 150,000), it also registers a much higher acceptance rate (compare 89% to 24%) and highly increased efficiency, as given by the median ESS (across all parameters) normalised by the number of PDEs evaluated (compare 0.35 to 0.04).

| Emulator | Acceptance rate | $\frac{\text{Median ESS}}{\text{no of PDEs}}$ |
|---|---|---|
| yes | 89% | 0.35 |
| no | 24% | 0.04 |

Table 2: Comparison of efficiency for models B and C in Tables 1 and 2 of the main paper, obtained with standard MCMC (model C) and MCMC with emulation – described in Section 7 (model B). Results for model B are based on 5000 iterations (i.e. PDE evaluations), and 150,000 for model C. We show the acceptance rate and the median ESS (across all parameters) normalised by the number of PDEs evaluated.

## References


[1] H. Akaike, Information theory and an extension of the maximum likelihood principle., in: Proc. 2nd International Symposium on Information Theory, Tsahkadsor, Armenia, USSR, Budapest: Akadémiai Kiadó, 1971, pp. 267—-281.

[2] G. Schwarz, Estimating the Dimension of a Model, Ann. Statist. 6 (2) (1978) 461–464.

[3] D. Spiegelhalter, N. Best, B. Carlin, A. Van Der Linde, Bayesian measures of model complexity and fit, Journal of the Royal Statistical Society: Series B (Statistical Methodology) 64 (4) (2002) 583–639.

[4] C. S. Bos, A comparison of marginal likelihood computation methods, in: COMPSTAT 2002: Proceedings in Computational Statistics, 2002, pp. 111–117.

[5] S. Watanabe, Asymptotic Equivalence of Bayes Cross Validation and Widely Applicable Information Criterion in Singular Learning Theory, J. Mach. Learn. Res. 11 (2010) 3571–3594.

[6] N. Friel, A. N. Pettitt, Marginal likelihood estimation via power posteriors, Journal of the Royal Statistical Society: Series B (Statistical Methodology) 70 (3) (2008) 589–607.

[7] N. Lartillot, H. Philippe, Computing Bayes Factors Using Thermodynamic Integration, Systematic biology 55 (2006) 195–207.

[8] A. E. Raftery, M. A. Newton, J. M. Satagopan, P. N. Krivitsky, Estimating the Integrated Likelihood via Posterior Simulation Using the Harmonic Mean Identity, in: In Bayesian Statistics, Vol. 8, University Press, 2007, pp. 1–45.

[9] A. Gelman, J. Hwang, A. Vehtari, Understanding Predictive Information Criteria for Bayesian Models, Statistics and Computing 24 (6) (2014) 997–1016.

[10] C. Rasmussen, C. Williams, Gaussian Processes for Machine Learning (Adaptive Computation and Machine Learning), The MIT Press, 2005.

[11] M. Ebden, Gaussian Processes: A Quick Introduction, ArXiv e-prints,`arXiv:1505.02965`.

[12] C. C. Holmes, L. Held, Bayesian auxiliary variable models for binary and multinomial regression, Bayesian Anal. 1 (1) (2006) 145–168.
[13] C. K. I. Williams, D. Barber, Bayesian classification with Gaussian processes, IEEE Transactions on Pattern Analysis and Machine Intelligence 20 (12) (1998) 1342–1351.
[14] T. V. Nguyen, E. V. Bonilla, Automated Variational Inference for Gaussian Process Models, in: Z. Ghahramani, M. Welling, C. Cortes, N. D. Lawrence, K. Q. Weinberger (Eds.), Advances in Neural Information Processing Systems 27, Curran Associates, Inc., 2014, pp. 1404–1412.
[15] M. Kuss, Gaussian Process Models for Robust Regression, Classification, and Reinforcement Learning, Ph.D. thesis, Technische Universität Darmstadt (2006).
[16] M. C. Kennedy, A. O'Hagan, Bayesian calibration of computer models, Journal of the Royal Statistical Society: Series B (Statistical Methodology) 63 (3) (2001) 425–464.
[17] K. P. Murphy, Machine learning: a probabilistic perspective, MIT Press, 2013.
[18] A. Gelman, J. Carlin, H. Stern, D. Dunson, A. Vehtari, D. Rubin, Bayesian Data Analysis, Third Edition, Chapman & Hall/CRC Texts in Statistical Science, Taylor & Francis, 2013.
[19] C. Andrieu, A. Doucet, Joint Bayesian Model Selection and Estimation of Noisy Sinusoids via Reversible Jump MCMC, IEEE Transactions on Signal Processing 47 (1999) 2667 – 2676.
[20] C. Robert, J.-M. Marin, Bayesian Core: A Practical Approach to Computational Bayesian Statistics, Springer Science & Business Media, 2007.
[21] H. Haario, E. Saksman, J. Tamminen, An adaptive Metropolis algorithm, Bernoulli 7 (2) (2001) 223–242.
[22] C. George, I. Edward, Explaining the Gibbs Sampler, The American Statistician 46 (3) (1992) 167–174.
[23] C. Rasmussen, Gaussian Processes to Speed up Hybrid Monte Carlo for Expensive Bayesian Integrals, Bayesian Statistics 7 7 (2003) 651–659.
[24] J. Christen, C. Fox, Markov Chain Monte Carlo using an Approximation, Journal of Computational and Graphical Statistics 14 (4) (2005) 795–810.
[25] C. Sherlock, A. Golightly, D. Henderson, Adaptive, Delayed-Acceptance MCMC for Targets With Expensive Likelihoods, Journal of Computational and Graphical Statistics 26 (2) (2017) 434–444.
[26] L. Mihaela Paun, M. Colebank, M. Umar Qureshi, M. Olufsen, N. Hill, D. Husmeier, MCMC with Delayed Acceptance using a Surrogate Model with an Application to Cardiovascular Fluid Dynamics, in: Proceedings of the International Conference on Statistics: Theory and Applications (ICSTA'19), 2019.
[27] P. Bratley, B. Fox, Algorithm 659: Implementing Sobol's Quasirandom Sequence Generator, ACM Trans. Math. Softw. 14 (1) (1988) 88–100.
[28] L. S. Bastos, A. O'Hagan, Diagnostics for Gaussian Process Emulators, Technometrics 51 (4) (2009) 425–438.
[29] L. M. Paun, M. U. Qureshi, M. Colebank, N. A. Hill, M. S. Olufsen, M. A. Haider, D. Husmeier, MCMC methods for inference in a mathematical model of pulmonary circulation, Statistica Neerlandica 72 (3) (2018) 306–338.
[30] C. Kreutz, A. Raue, D. Kaschek, J. Timmer, Profile likelihood in systems biology, The FEBS Journal 280 (11) (2013) 2564–2571.
[31] R. Kass, B. Carlin, A. Gelman, R. Neal, Markov Chain Monte Carlo in Practice: A Roundtable Discussion, The American Statistician 52 (2) (1998) 93–100.
